# Tissue-Specific miRNAs Regulate the Development of Thoracic Aortic Aneurysm: The Emerging Role of KLF4 Network

**DOI:** 10.3390/jcm8101609

**Published:** 2019-10-03

**Authors:** Stasė Gasiulė, Vaidotas Stankevičius, Vaiva Patamsytė, Raimundas Ražanskas, Giedrius Žukovas, Žana Kapustina, Diana Žaliaduonytė, Rimantas Benetis, Vaiva Lesauskaitė, Giedrius Vilkaitis

**Affiliations:** 1 Institute of Biotechnology, Vilnius University, LT-10257 Vilnius, Lithuania; stase.gasiule@bti.vu.lt (S.G.); raimundas.razanskas@bti.vu.lt (R.R.); 2 Institute of Cardiology, Lithuanian University of Health Sciences, LT-50103 Kaunas, Lithuania; vaiva.patamsyte@lsmuni.lt (V.P.); rimantas.benetis@kaunoklinikos.lt (R.B.); vaiva.lesauskaite@lsmuni.lt (V.L.); 3 Department of Cardiac, Thoracic and Vascular Surgery, Lithuanian University of Health Sciences, LT-50103 Kaunas, Lithuania; giedrews@yahoo.com; 4 Thermo Fisher Scientific Baltics, LT-02241 Vilnius, Lithuania; zana.kapustina@thermofisher.com; 5 Department of Cardiology, Lithuanian University of Health Sciences, LT-50161 Kaunas, Lithuania

**Keywords:** aortic disease, aneurysm, miRNA, TGF-β pathway, KLF4, synthetic phenotype

## Abstract

MicroRNAs (miRNAs) are critical regulators of the functional pathways involved in the pathogenesis of cardiovascular diseases. Understanding of the disease-associated alterations in tissue and plasma will elucidate the roles of miRNA in modulation of gene expression throughout development of sporadic non-syndromic ascending thoracic aortic aneurysm (TAA). This will allow one to propose relevant biomarkers for diagnosis or new therapeutic targets for the treatment. The high-throughput sequencing revealed 20 and 17 TAA-specific miRNAs in tissue and plasma samples, respectively. qRT-PCR analysis in extended cohort revealed sex-related differences in miR-10a-5p, miR-126-3p, miR-155-5p and miR-148a-3p expression, which were the most significantly dysregulated in TAA tissues of male patients. Unexpectedly, the set of aneurysm-related miRNAs in TAA plasma did not resemble the tissue signature suggesting more complex organism response to the disease. Three of TAA-specific plasma miRNAs were found to be restored to normal level after aortic surgery, further signifying their relationship to the pathology. The panel of two plasma miRNAs, miR-122-3p, and miR-483-3p, could serve as a potential biomarker set (AUC = 0.84) for the ascending TAA. The miRNA-target enrichment analysis exposed TGF-β signaling pathway as sturdily affected by abnormally expressed miRNAs in the TAA tissue. Nearly half of TAA-specific miRNAs potentially regulate a key component in TGF-β signaling: TGF-β receptors, SMADs and KLF4. Indeed, using immunohistochemistry analysis we detected increased KLF4 expression in 27% of TAA cells compared to 10% of non-TAA cells. In addition, qRT-PCR demonstrated a significant upregulation of ALK1 mRNA expression in TAA tissues. Overall, these observations indicate that the alterations in miRNA expression are sex-dependent and play an essential role in TAA via TGF-β signaling.

## 1. Introduction

Thoracic aortic aneurysms (TAAs) are usually silent and therefore deadly if not detected and repaired on time [1]. Most of them are affecting the root or ascending aorta [2]. TAA is categorized as syndromic (Marfan, Loyes-Dietz, Ehlers-Danlos, etc.), familial non-syndromic and sporadic [3]. The incidence of TAA is permanently increasing and remains much higher in males than females [4]. Similar trends are observed in hospital admissions for TAA [5]. The aortic diseases are more common in males but the outcome is worse in female patients, although reasons for sex differences are unknown [6]. Therefore, further investigation of molecular mechanisms for these differences are required as well [7]. Vascular smooth muscle cells (VSMC) have been shown to possess a natural plasticity to switch between contractile and synthetic phenotypes in order to repair small vascular injuries [8]. For the last few decades, VSMC dedifferentiation has been recognized as one of the key processes involved in arterial maintenance and development of vascular diseases [9]. This led to the identification of various regulators of VSMC phenotype including a transcription activator myocardin (*MyoCD*) [10], transcription factor Krüppel-like factor 4 (*KLF4*) [11], and components of a transforming growth factor beta (*TGF-β*) signalling pathway [12]. During the formation of TAA, VSMCs are thought to lose their contractile ability and start secretion of various extracellular matrix proteins and their inhibitors, but the mechanism of this phenotypic shift remains unknown. 

Recent studies have focused on the emerging epigenetic regulation of gene expression and the short non-coding microRNAs (miRNAs) involved in post-transcriptional regulation of a target messenger RNA (mRNA) [13]. MiRNAs have been implicated in the pathogenesis of various cardiovascular diseases [14] and show the potential to be utilised as biomarkers in diagnosis, prognosis, and selection of treatment [15]. Over the last decade a variety of miRNAs have been identified in the regulation of VSMC phenotype [16,17,18] some of which have been associated with the formation of TAA [19,20]. The majority of miRNAs association studies were done using PCR or microarray techniques [21] and only a fraction of miRNAs have been validated in plasma samples [22]. However, to date, the global high-throughput miRNA sequencing data of TAA tissue is still missing. Furthermore, most of miRNA-related mechanistic insights of TAA development are made using cell cultures or knock-out animal models which only mildly represents human disease and could further lead to misinterpretation of biological processes occurring in human tissue *in vivo* [23].

A multidimensional approach is needed in order to uncover the complex mechanisms occurring in the human aortic wall during the formation of TAA and to evaluate the possibility of using circulating miRNAs as biomarkers for the development of the disease. In the present study, we aimed to profile miRNA changes in TAA tissue and blood plasma samples and to assess their role in the pathogenesis of the disease as well as to evaluate their potential to be used as biomarkers. Using high-throughput miRNA sequencing we identified 20 and 17 differentially expressed miRNAs in TAA tissue and TAA plasma samples compared to non-TAA specimens, respectively. Deregulation of selected miRNAs in TAA samples were further confirmed by qRT-PCR analysis, thus verifying reliability of miRNA-Seq results. A subsequent pathway enrichment analysis of miRNA target genes revealed significant relationship between nearly half of dysregulated miRNAs and TGF-β signalling pathway. Finally, we for the first time showed accumulation of KLF4, a master regulator of VSMC differentiation state, in TAA tissue obtained from patients. Altogether our results define potential candidates for TAA diagnostic biomarkers and provide new insights in regulatory miRNA-related mechanisms of TAA development.

## 2. Materials and Methods

### 2.1. Patient Samples

All experimental procedures using human tissue and plasma samples conform to the principles outlined in the Declaration of Helsinki and were approved by Kaunas Regional Biomedical Research Ethics Committee (Nr. P2-BE-2-12/2012). 

The study included 40 patients with sporadic non-syndromic ascending thoracic aorta aneurysm (TAA group). Exclusion criteria were severe atherosclerosis showing calcified or ulcerating plaques of the ascending aorta, aortitis, phenotypic characteristics of the known genetic disorders such as Marfan, Ehlers Danlos and other syndromes. The diagnosis was confirmed by two-dimensional thoracic aorta echocardiography according to the 2014 ESC guidelines on the diagnosis and treatment of aortic diseases. Echocardiography was performed at the Department of Cardiology, Lithuanian University of Health Sciences (LUHS). TAA group included patients (*n* = 23) who underwent aortic reconstruction surgery at the Department of Cardiac, Thoracic and Vascular Surgery, LUHS and non-operated patients (*n* = 17) with ascending aorta aneurysm. 

Study subjects without TAA (non-TAA group) included i) heart transplantation donors (*n* = 6), ii) patients who underwent isolated coronary artery bypass graft surgery (CABG) (*n* = 72) and iii) healthy volunteers (*n* = 10). All healthy volunteers were screened using two-dimensional transthoracic echocardiography to ensure the ascending aorta was not dilated. Detailed preparation of patients’ tissue and plasma samples can be found in the Supplementary Methods.

### 2.2. Study Design

Study subjects (*n* = 32) selected for miRNA expression profiling in aortic tissue consisted of surgical TAA patients (*n* = 8), donors (*n* = 4) as well as CABG patients (*n* = 2). miRNA expression profiling in plasma was done in samples from surgical TAA patients (*n* = 7) before and 3 months after the aortic surgery (denoted as operated, *n* = 4), respectively. Seven volunteers without health complaints (n= 7) were used as non-TAA controls. Clinical and demographic characteristics of the groups are summarized in Table 1.

Validation group for miRNA expression in aortic tissue consisted of TAA surgical patients (*n* = 17), donors and CABG patients (*n* = 35). For the miRNA validation in plasma samples, we were able to collect larger TAA group (*n* = 28) and non-TAA group (*n* = 34) which consisted of healthy volunteers and CABG patients. Clinical and demographic characteristics of each validation group are presented in Supplementary Table S1. A significant difference in ascending aortic diameter (*p* < 0.001) was observed between TAA patients and non-TAA in both miRNA profiling groups supporting the selection criteria. Patients with bicuspid aortic valve were predominant in both TAA groups (*p* < 0.001) compared with non-TAA group. 

Total RNA isolation, cDNA library sequencing, and miRNA-Seq differential and functional analysis are described in detail in Supplementary Methods. miRNA-Seq data are available at GEO database using accession number GSE122266. Validation of miRNA-Seq data was performed as described previously [24] and detailed qRT-PCR and immunohistochemistry analysis are depicted in Supplementary Methods.

## 3. Results

### 3.1. Differential miRNA Expression Analysis in TAA Tissue and Blood Plasma Samples

**Figure 1:**
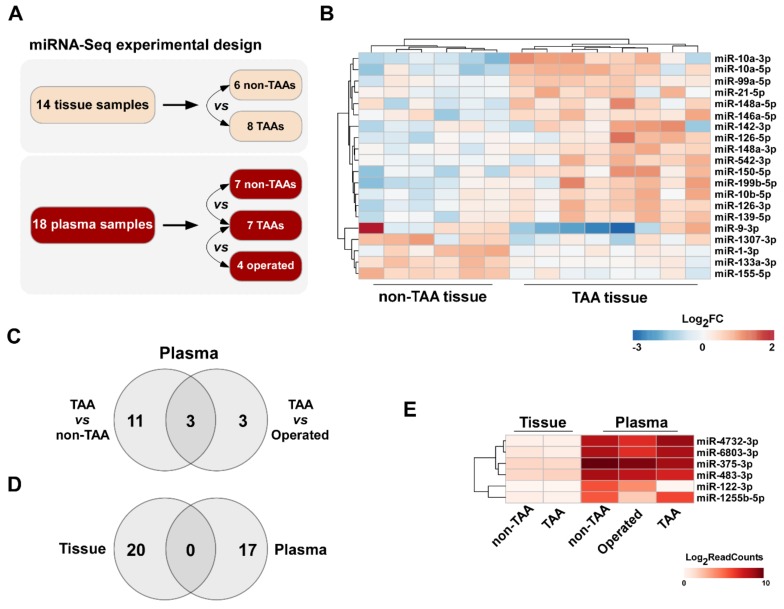
Differential miRNA expression analysis in TAA tissue and plasma samples using high-throughput RNA sequencing. (**A**) Schematic diagram of miRNA-Seq experiment. (**B**) Heat map showing a total of 20 miRNAs differentially expressed (fold change, FC > 1.5, *p* < 0.05, normalized read count average, RC > 10) in TAA tissue samples (*n* = 8) compared to normal aorta tissue (*n* = 6). Red color indicates upregulated log-transformed expression level ratios of corresponding miRNAs, blue – downregulated; (**C**) Venn’s diagram showing the number of differentially expressed miRNAs (FC > 1.5, *p* ≤ 0.05 and RC > 20) in TAA plasma samples (*n* = 7) compared to non-aneurysmal group (*n* = 7) and plasma samples obtained 3 months after aortic reconstructive surgery (*n* = 4); (**D**) Venn’s diagram demonstrating the number of differentially expressed miRNAs in TAA tissue and plasma samples; (**E**) Heat map demonstrating the expression of six miRNAs, which were significantly deregulated in TAA plasma samples, but were almost absent in TAA tissue samples. Color intensity indicates log-transformed normalized read counts of corresponding miRNA.

In order to determine miRNAs which expression levels are potentially deregulated in aorta tissue and blood plasma during the formation of TAA, in the present study we evaluated miRNA expression profiles in a learning set of patient tissue and plasma samples (*n* = 32) using Illumina high-throughput miRNA sequencing platform (Table 1; Figure 1A). The overview of miRNA-Seq experimental design and data quality is depicted in Supplementary Results. miRNA-Seq data analysis revealed a total of 20 differentially expressed miRNAs (selection criteria were fold change ≥ 1.5, *p* value < 0.05 and base mean higher than 10) in TAA tissue samples compared to non-TAA group (Table 2), among which the majority (15 of 20 miRNAs) were upregulated (Table 3). A detailed differential miRNA-Seq data evaluation of each sample assessed in the present study is depicted in Supplementary File 1 and 2. A heat map of expression signature for these dysregulated miRNAs in all 14 samples clearly clustered aorta tissues according to the presence or absence of aneurysm (Figure 1B).

Using the same workflow of miRNA-Seq analysis, we found 14 differentially expressed miRNAs in TAA patient plasma samples compared to non-TAA group. Out of these, 3 were upregulated and 11 were downregulated (Table 2 and Table 4). Next, to determine alterations after the removal of aneurysm, we compared the miRNA expression levels between TAA plasma samples collected before and 3 months after aortic surgery. This analysis led to the detection of six differentially expressed miRNAs (Table 4; Supplementary Figure S1). Remarkably, the expression of three of TAA-specific plasma miRNAs, miR-1255b-5p, miR-122-3p and miR-23b-5p, returned to near non-TAA levels after the operation. Finally, to identify the most significantly dysregulated miRNAs in TAA plasma samples, we pooled data from both non-aneurysmal (non-TAA and TAA samples collected after the surgery) cohorts and compared to TAA group. The differential analysis determined ten differentially expressed miRNAs revealing the greatest fold change for miR-122-3p. Thus, the overall evaluation of miRNA expression changes in plasma samples discovered a total of 17 differentially expressed miRNAs in TAA samples compared to non-TAA samples, samples collected after aortic surgery or both groups of samples (Figure 1C). 

Surprisingly, a pattern of aneurysm-related alterations in plasma’s miRNA profiles showed no resemblance to the tissue set. Indeed, none of the differentially expressed miRNAs overlapped in Venn’s diagram (Figure 1D). Moreover, the expression levels of the six significantly deregulated miRNAs in TAA plasma samples, miR-4732-3p, miR-6803-3p, miR-375-3p, miR-483-3p, miR-122-3p and miR-1255b-5p, were negligible in aortic tissue samples (Figure 1E).

### 3.2. Validation of Selected miRNAs in TAA Tissue and Plasma Samples by qRT-PCR

**Figure 2:**
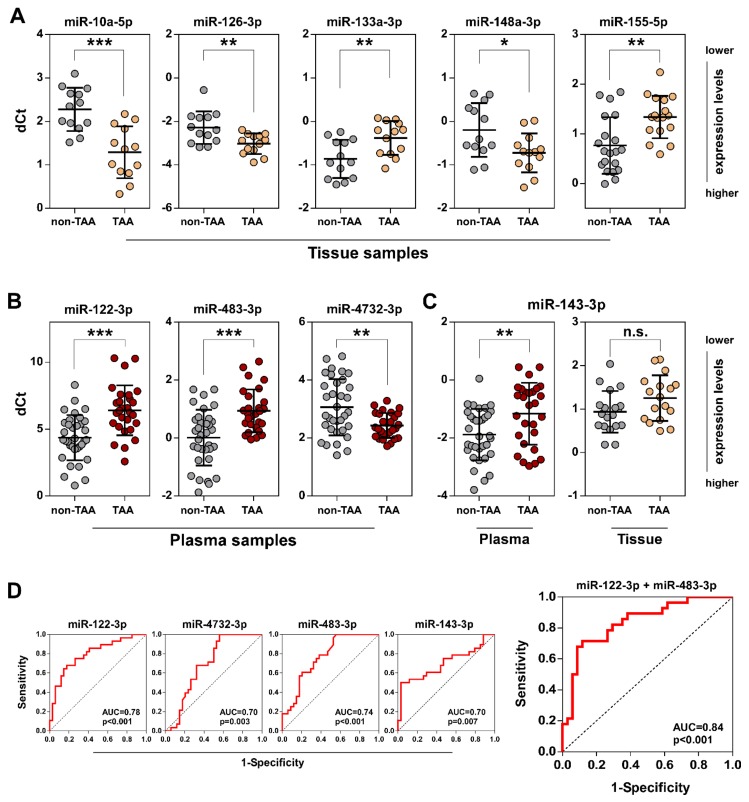
Validation of differentially expressed miRNAs in TAA tissue and plasma samples by qRT-PCR. qRT-PCR analysis was used for the comparison of relative miRNA expression levels between non-TAA and TAA groups in tissue (**A**) and plasma (**B**) both types (**C**) of samples. The cycle threshold (Ct) values of observed miRNAs were normalized to miR-152-3p and miR-185-5p for tissue and plasma samples, respectively, which were revealed as the most reliable endogenous controls according to miRNA-Seq data. Lines within boxes indicate relative miRNA expression median values; whiskers—5–95 percentile of the relative miRNA expression values. Significance between each group was evaluated using Student’s t test and is shown as follows: n.s.—not significant; * *p* < 0.05; ** *p* < 0.01 and *** *p* < 0.001. (**D**) Diagnostic ROC curve analysis showing sensitivity and specificity of mir-122-3p, mir-483-3p, mir-4732-3p and mir-143-3p selected circulating miRNAs or the combination of mir-122-3p and mir-483-3p together. AUC denotes area under the ROC curve.

In order to corroborate the RNA sequencing-based predictions, we performed qRT-PCR analysis to examine the abundance of five selected miRNAs (mir-10a-5p and miR-155-5p exhibited the greatest up/down fold changes; miR-126-3p, mir-133a-3p and miR-148a-3p were implicated in TGF-β signaling routes, see below) in the independent group of 37 samples containing 20 non-TAA and 17 thoracic aortic aneurysm tissues (Supplementary Table S1). Our analysis validated the up- and downregulated expression of miR-10a-5p, miR-126-3p, miR-133a-3p and miR-155-5p, respectively, in the sex-undivided set of TAA tissue samples compared to the non-TAA group (Figure 2A, Supplementary Figure S2). Whereas, the difference in miR-148a-3p expression levels was significant among the groups only when stratifying by sex (*p* = 0.0203 for a male patient set). Interestingly, a significantly greater differential expression of miR-126-3p (*p* = 0.0062 vs. *p* = 0.0225 for sex-undivided set), miR-155-5p (*p* = 0.0003 vs. *p* = 0.0017) and miR-10a-5p (*p* = 0.0001 vs. *p* = 0.0002), except miR-133a-3p (*p* = 0.0068 vs. *p* = 0.0031), also was observed between 13 TAA and 13 normal aorta tissue samples from male patients showing sex-related miRNA expression variances in the TAA tissue. Because of the scarce representation of female samples (7 non-TAA vs. 4 TAA) the extent of involvement of these miRNAs in the thoracic aneurysm formation in female patients requires further analysis.

In contrast, the evaluation of the miRNA expression levels in 62 plasma samples (34 non-TAA vs. 28 TAA) demonstrated that all selected miRNAs, miR-4732-3p, miR-483-3p and miR-122-3p, exhibited statistically significant expression changes in TAA plasma samples compared to non-TAA group showing a good reliability of our miRNA-Seq data (Figure 2B). Of those, the difference of miR-122-3p expression levels was the most significant (*p* < 0.0001) between two plasma sample groups.

RNA-Seq analysis displayed that the difference of miR-143-3p expression was not statistically significant in TAA tissue samples compared to non-TAA. Consistently with this observation, a significant downregulation (*p* = 0.0051) was observed only in TAA plasma samples (Figure 2C) despite the decrement of miR-143-3p expression level in both TAA specimen groups compared to non-TAA group.

The diagnostic sensitivity and specificity of selected plasma circulating miRNAs which could serve as potential biomarkers of TAA was examined by ROC curve analysis (Figure 2D). The results demonstratostic accuracy (AUC = 0.78, *p* < 0.001). Moreover, a combined analysis of miR-122-3p and miR-483-3p miRNAs showed even better diagnostic discrimination (AUC = 0.84, *p* < 0.001) indicating that these miRNAs could be applied as TAA biomarkers.

Finally, the statistical analysis showed no significant correlations between selected differential miRNAs, miR-10a-5p, miR-126-3p, mir-133a-3p miR-155-5p, miR-148a-3p, miR-122-3p, miR-483-3p, miR-4732-3p, miR-143-3p and chosen patient characteristics such as patients’ age, aorta diameter, bicuspid (BAV)/tricuspid aortic valves (TAV)-associated aneurysms (data is not shown).

On the other hand, we revealed a significant moderate positive correlations between expression level of miR-126-3p and miR-148a-3p (*R* = 0.67) or miR-10a-5p (*R* = 0.67), miR-148a-3p and miR-10a-5p (*R* = 0.49), miR-133a-3p and miR-155-5p (*R* = 0.67) (Supplementary Table S2). Meanwhile, in plasma samples, the strongest correlation was observed between the expression of miR-122-3p and miR-483-3p (*R* = 0.65) (Supplementary Table S3).

### 3.3. Functional Analysis of miRNA Target Genes Involved in TAA Development

**Figure 3:**
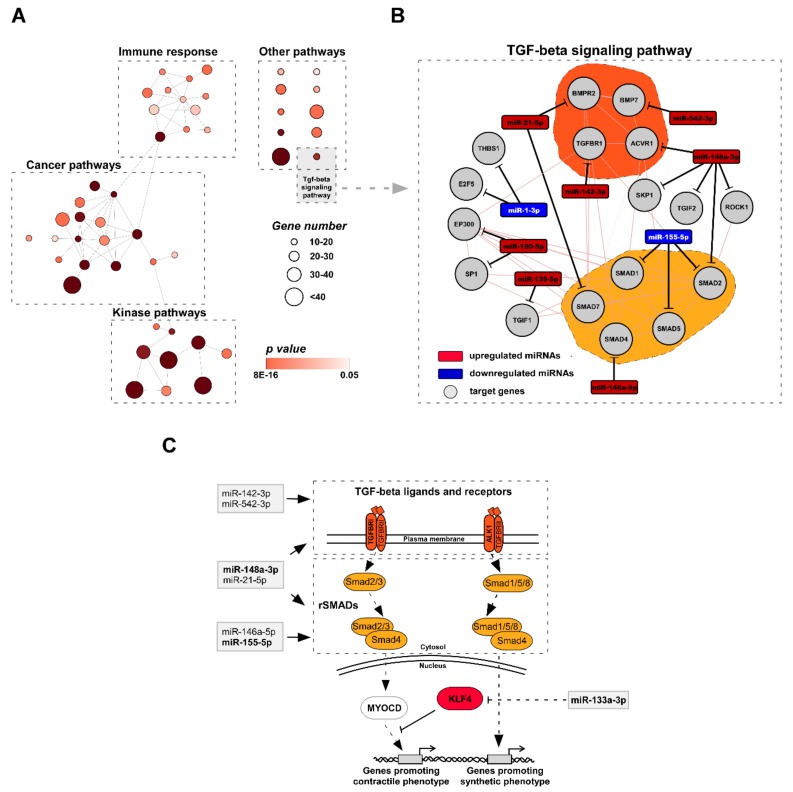
Functional analysis of target genes of miRNAs dysregulated in TAA. (**A**) Network analysis of 48 KEGG categories specified three clusters of closely related categories including immune response, cancer, kinase signaling pathways and ten separate groups that were not significantly associated with any other category. *TGF-β* signaling pathway is included in a grey box. The size of node represents gene number in particular, KEGG category, the node color – the significance level value of particular KEGG category. Edges indicate a statistically significant association between categories. (**B**) Expanded molecular network of miRNAs and their potential target genes involved in *TGF-β* signaling pathway. Grey nodes denote target genes, red and blue – upregulated and downregulated miRNAs, respectively. Dark orange area covers *TGF-β* ligands and receptors; light orange – regulatory *SMAD*s (r*SMAD*s). (**C**) Simplified hypothetical schema of *TGF-β* signal transduction in TAA tissue cells. miRNAs, which were differentially expressed in TAA tissue (grey boxes), could potentially disturb *TGF-β* signaling by targeting *TGF-β* ligands, receptors or r*SMAD*s leading to dysregulation of *MyoCD*–*KLF4* transcription regulator axis and further TAA progression.

Next, we performed KEGG pathway enrichment analysis of dysregulated miRNA target genes to unravel the miRNA-mediated biological processes associated with TAA development. To provide the best set of relevant candidates for the bona fide miRNA-mRNA interactions, we evaluated combined scores from eight different miRNA target site prediction databases (see details in Methods). The examination identified 1133 target genes (exceeding combined score threshold value of 4) for group consisting of miRNAs which were differently expressed in TAA tissue (Supplementary File 3). The subsequent pathway enrichment analysis of the defined miRNA target sets revealed 48 KEGG categories significantly enriched in targeted genes (> 15 target genes in functional category, FDR < 0.05; Supplementary Table S4). In order to visualize the interconnection between signaling pathways regulated by miRNAs, we generated KEGG pathway network using Cytoscape plugin ClueGo. The network analysis clearly exposed three large functional clusters of KEGG categories closely related to immune response, cancer development and kinase signaling pathways, while any significant association of the remaining ten pathways to any other category was absent (Figure 3A, Supplementary Table S5). Transforming growth factor beta (*TGF-β*) signaling pathway, which plays a key role during aorta development and subsequent remodeling, was represented among these categories. To make a more detailed assessment of the miRNA-target interaction network, we additionally introduced target genes which were significantly related to differentially expressed miRNAs (target score > 4).

The expanded analysis revealed 17 target genes which could be potentially regulated by 9 out of 20 of miRNAs differentially expressed in TAA tissue (Figure 3B). Furthermore, our results defined two groups of genes sharing the similar functions which could be potentially affected by miRNAs - (i) *TGF-β* receptors and ligands and (ii) regulating *SMAD*s (r*SMAD*s) (Figure 3C, grey boxes). As shown above, the differential expression of two of them, miR-148a-3p and miR-155-5p, were additionally confirmed by qRT-PCR analysis. Moreover, miR-133a-3p, which was significantly downregulated in TAA tissue samples, has been previously described as a prominent indirect downregulator of Kruppel-like factor 4 (*KLF4*) [25]. According to these findings, we hypothesized that the miRNAs related to TAA could contribute significantly to critical changes in tissue remodeling in diseased aorta through of *TGF-β* signaling pathway: (i) leading to the functional dysregulation of the key regulators, *KLF4* and/or *MyoCD*, which determine the differentiation state of aorta smooth muscle cells (Figure 3C); (ii) the altered signaling balance between *TGF-β* receptors, ligands and r*SMAD*s could provoke alternative *MyoCD*-independent *TGF-β* signaling routes which could boost TAA development.

### 3.4. Number of VSMCs Expressing KLF4 Dramatically Increases during TAA Development

**Figure 4:**
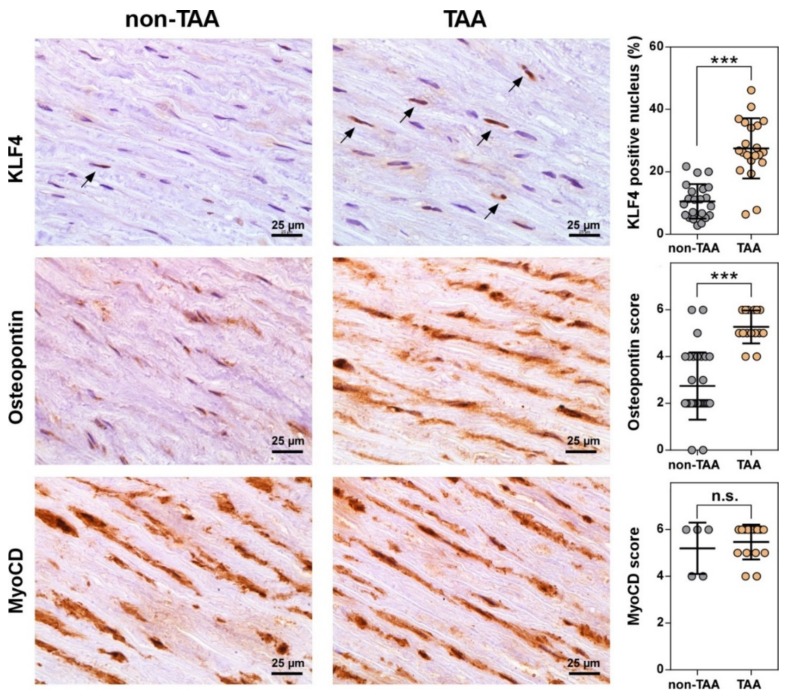
Immunohistochemical (IHC) analysis of KLF4, MyoCD, and osteopontin expression in non-TAA and TAA tissue samples. The abundance of proteins was examined by immunostaining and visualized with diaminobenzidine (brown). The sections were counterstained with hematoxylin (blue). Histological quantification of KLF4 was performed by counting KLF4 positive cell nucleus (black arrows; *n* = 43), whereas osteopontin (*n* = 46) and MyoCD (*n* = 20) by IHC score (graphs in right panel). Lines within boxes indicate KLF4 positive nucleus mean or MyoCD and osteopontin IHC score median values, whiskers – 5-95 percentile of KLF4 positive nucleus or MyoCD and osteopontin IHC score values. The histological data were assessed using Student’s t test (for KLF4) or non-parametric Mann-Whitney U test (for MyoCD and osteopontin). The significance between each group is shown as follows: n.s.—not significant; * *p* < 0.05; ** *p* < 0.01, *** *p* < 0.001.

To further explore the compelling connection of the reprogramed miRNA network with *TGF-β* signaling pathway, we assessed the mRNA expression levels of *TGF-β* receptors, *TGFBR1* and *ALK1* (also known as *ACVRL1*), and transcription factors, *MyoCD* and *KLF4*, in non-TAA (n=21) and TAA (*n* = 17) tissue samples (Supplementary Figure S3A). We observed a relevant elevation of *ALK1* gene transcription in TAA tissues (*p* = 0.0244) compared to non-TAA group of normal aortas but found no significant difference in cellular mRNA levels of *TGFBR1*, *KLF4*, and *MyoCD*. It is noteworthy that we revealed a moderate positive correlation between the changes in expression of *ALK1*, miR-10a-5p, miR-126-3p and miR-148a-5p (Supplementary Table S6; Supplementary Figure S4) suggesting a putative biological relationship between these miRNAs and *TGF-β* signaling pathway. Thus, the obtained data pointed out to a weak regulation of studied genes, except *ALK1*, on transcription or mRNA decay level. However, it has been reported that many human miRNAs control post-transcriptional processes at the protein translation stage [26]. Therefore, we further evaluated the protein expression levels of the selected genes in non-TAA and TAA tissue samples using immunohistochemical analysis (IHC) (Figure 4; Supplementary Figure S3B). The IHC analysis revealed higher expression of *ALK1* both in normal and TAA tissues (IHC score median = 4), whereas the expression levels of *TGFBR1* remained lower in both aortic sample groups (IHC score median = 2). However, the difference of expression levels of both *TGF-β* receptors remained insignificant between TAA and non-TAA specimens (Supplementary Figure S3B). In contrast, although the overall expression levels of *KLF4* were low in both groups of aortic samples, we detected a significant three-fold accumulation (*p* = 0.0037) of *KLF4* positive cells in TAA tissues compared to non-TAA group (Figure 4). Meanwhile, IHC analysis strongly supported upregulated expression of osteopontin (*p* = 0.0311), which is indicating shift of vascular smooth muscles from contraction to synthetic phenotype and is a positive marker of aortic aneurysms [27,28]. Finally, despite IHC results displaying high levels of *MyoCD* in both groups of samples (IHC score median = 6), a significant expression difference was absent between groups (Figure 4, lower panel).

## 4. Discussion

Aneurysm is one of the most frequent diseases of the aorta [29]. The aortic aneurysms rarely cause any symptoms and thereby are commonly diagnosed incidentally. Consequently, the rupture or dissection of the aneurysm leads to lethal outcomes in over 15000 cases annually in the USA only [30]. Classification of aortic aneurysms is based on the anatomic location, with thoracic aortic aneurysms involving the ascending and descending aorta and abdominal aortic aneurysm [31]. The ascending aorta is derived from distinct embryonic origin defining some specific pathological aspects of aneurysms appearing in different locations [32]. The distinct disease entities at the molecular level may be regulated by specific, at least partially, miRNA networks. Previous studies have demonstrated that miRNAs play key roles during the formation of AAA by dysregulating VSMC homeostasis and extracellular matrix (ECM) composition or inducing vascular inflammatory response [33,34]. However, a global high-throughput miRNA-sequencing data of TAA tissue and plasma was still missing, despite some experimental data obtained by miRNA microarrays [19,20,35].

### 4.1. miRNA Expression Patterns in Tissues May Be Influenced by Aneurysmal Location and Sex

In the present study we determined a total of 20 miRNAs which were differentially expressed in ascending TAA tissues compared to non-aneurysmal group. A qRT-PCR testing further validated the differential expression of four selected miR-10a-5p, miR-133a-3p, miR-126-3p, miR-155-5p and miR-148a-3p in a larger set of independent samples supporting the reliability of miRNA-Seq results.

Despite a partial overlap (miR-126-3p, miR-155-5p and mir-133a-3p appeared to be involved in AAA [22,29,30]), our results confirmed previous assumptions indicating a quite distinct miRNA expression patterns between TAA and AAA tissues. This might be explained by different pathophysiological mechanism for ascending aneurysm development in comparison to AAA. The latter is most commonly caused by atherosclerosis [31]. In agreement with the present study, the dysregulated expression of miR-133a-3p, miR-126-3p and miR-155-5p has been previously associated with TAA [32]. However, the pathophysiological functions of selected miRNAs in TAA formation and how they modulate disease progression remain poorly understood. A significant upregulation of miR-155-5p was identified in various cardiovascular diseases including AAA and was linked to the inflammatory response in aortic wall. It was demonstrated that expression of miR-155 correlated with inflammatory macrophage response and extracellular matrix destruction in AAA model mice [33]. On the contrary, we identified miR-155-5p as the most strongly downregulated miRNA in TAA tissue. It might be explained by the absence of advanced atherosclerosis leading to inflammatory response in the studied ascending aorta samples obtained during aortic reconstruction. Meanwhile, vascular endothelium specific miR-126-3p is required for the maintenance of vascular integrity and endothelial cell homeostasis [34]. Reduced in proliferating VSMC miR-133a-3p switch on transcription factor *Sp1*, which activates *KLF4*, thus promoting synthetic phenotype [25,35]. miR-10a-5p, one of the most upregulated miRNAs in this study, and miR-148a-3p previously were not related to TAA. It was reported that increased miR-10a-5p expression leads to VSMC differentiation from embryonic stem cells through repression of histone deacetylase *HDAC4* [36]. Thus, we inferred that miR-10a-5p may be a potential modulator of VSMC phenotype as well.

We identified a positive correlation between expression levels of miR-126-3p and newly predicted TAA-related miR-10a-5p and miR-148a-3p (Supplementary Figure S4) indicating a possible functional or regulatory link between these miRNAs. Therefore, further studies of the molecular impact of miR-148a-3p and miR-10a-5p on TAA development is of interest.

An evaluation of sex-dependence revealed that miR-148a-3p varied significantly only in male TAA cohort (Supplementary Figure S1). Furthermore, miR-126-3p, miR-155-5p and miR-10a-5p expression changes were more statistically significant in male TAA patients. Notably, the incidence of TAA is more prevalent in males than females [4]. Moreover, previous reports emphasized relevant sex differences in the pathology of TAA, although underlying molecular mechanisms are unknown. Accordingly, aortic dilation rate was more than 3 times greater in women than in men [6]. This observation was associated with different levels of metalloproteinases *MMP2* and *MMP9*, inhibitory enzymes *TIMP1* and *TIMP2* and overall aortic stiffness highlighting a different ECM remodeling in female aortas [37]. Altogether, these findings suggest that sex-dependent physiological differences could be associated with different changes of miRNA levels in male aorta tissue during TAA development. Otherwise, a deregulation of miRNAs in sex-dependent manner could promote distinct pathways leading to different TAA progression and pathology rates in male patients.

### 4.2. Circulating miRNA Profile Does Not Match to Aneurysmal Signature of TAA Tissues

Herein we revealed 17 differentially expressed miRNAs in the TAA patients’ plasma samples compared to the non-TAA group. The combination of two of them, miR-122-3p and miR-483-3p, allowed to distinguish TAA patients from non-TAA subjects suggesting a novel set of prognostic biomarkers for TAA non-invasive diagnostic. Notably, the majority of altered miRNAs were associated with TAA for the first time, and so far, have not been previously identified in AAA samples [22]. 

Suprisingly, the pattern of aneurysm-related alterations in TAA patients’ plasma miRNA profile does not overlap with the tissue set (Figure 1D). Moreover, the expression of six miRNAs, which were among the most strongly deregulated in TAA plasma samples, was almost absent in TAA tissue. We can speculate that miRNA expression changes in TAA patient’s plasma could be evoked by a complex physiological organismal response to the aortic aneurysm development passed by circulating miRNAs that are essential vehicles for organ-to-organ cross-talk between liver, pancreas, muscle, immune and endothelial cells [38]. For instance, miR-122, the most strongly downregulated miRNA in this study, is a key factor in liver development, homeostasis and metabolic functions [39]. The downregulation of miR-122 in the liver cells correlates with hepatic pathology [40], which could be further associated with metabolic syndrome and cardiovascular diseases [41]. In blood plasma, the downregulation of miR-122 was previously associated with other cardiovascular diseases including bicuspid aortic valve, myocardial infarction and cardiac arrest [42,43,44]. Thus, it seems that the dysregulation of circulating miR-122, highlighted in our study, is not TAA tissue-associated directly but rather is determined by response to the TAA. Meanwhile, an altered expression of mir-483-3p was associated with endothelial cell response to vascular injury [45]. 

### 4.3. miRNA Target Analysis Reveals KLF4 As a Key Factor for the TAA Development in vivo

Using bioinformatics approach, we exposed 48 KEGG pathways enriched in genes targeted by differentially expressed miRNAs in TAA tissue cells. Functional categories annotated by KEGG displayed overlapping among target genes which were mainly associated with the immune response, cancer, and kinase activity processes. We emphasized “*TGF-β* signaling pathway” as individual pathways of highest importance which could be involved in TAA development (Supplementary Table S5). Indeed, about half of differentially expressed miRNAs have predicted targets in 17 genes involved in *TGF-β* pathway. These miRNAs could interfere with the signal transduction by affecting two principal groups of target genes - *TGF-β* ligands/receptors and regulatory *SMAD*s (Figure 3C). Based on these findings, we hypothesized that the mis-expressed miRNAs could contribute significantly to critical changes in diseased aorta via alterations in components of *TGF-β* signaling pathway. It can lead to the functional dysregulation of the key downstream regulators, *KLF4* and/or *MyoCD*. On the other hand, the deregulated balance between *TGF-β* receptors, ligands and r*SMAD*s might trigger alternative *MyoCD*-independent *TGF-β* signaling routes promoting further TAA development [46]. As shown in Figure 3, a group of *TGF-β* receptors/r*SMAD*s-associated miRNAs was mis-regulated in TAA tissues. Previous reports revealed that deficiency of *SMAD4* and *TGFBR2* in VSMCs induced aortic dilation in TAA mice model indicating that the imbalance of TGF-β receptors and r*SMAD*s could promote TAA progression [47,48]. On the other hand, the overexpression and over-activation of *SMAD2* was *TGF-β* signaling independent in TAA tissue samples suggesting a functional dissociation between the Smad2 activation and activity of the *TGF-β* receptors [47,49]. A signaling switch from canonical *TGFBRI*/*Smad2*-dependent to *ALK1*/*Smad1/5/8* signaling was shown to activate genes related to synthetic VSMC phenotype in mice model [50]. In addition, previous report indicated that the differentiation state of VSMCs is controlled by miR-26a via suppression of *TGF-β* signaling molecules [51] demonstrating a link between the dysregulated miRNA expression and aberrant *TGF-β* signaling during TAA development. 

Herein we demonstrated a significant upregulation of *ALK1* mRNA expression in TAA tissue cells compared to non-TAA. The expression of *ALK1* positively correlated with the levels of miR-126-3p, mir-10a-5p and miR-148a-3p indicating a functional connection between these miRNAs and *TGF-β* signaling. A follow-up IHC analysis revealed no significant changes in *ALK1* protein level in TAA tissues. However, the discrepancy between mRNA and protein assessments could be related to insufficient sensitivity of the immunohistochemistry approach that hamper the quantification of the modest changes at the protein level. Nevertheless, the main advantage of IHC analysis compared to other methods evaluating total levels of gene expression is the feasibility to visualize precisely the protein localization in individual cells of tissue. Thereby, the number of cells strongly expressing *KLF4* factor in the nucleus was shown to be nearly three-fold higher in TAA tissues compared to non-TAA (27% vs. 10%). Meanwhile, myocardin expression level appeared to be similar, although the precise estimation of the nuclear protein is encumbered by rather high myocardin abundance in cytoplasm. Thus, our *in vivo* data indicate that upregulation of *KLF4* does not directly abrogate the myocardin expression but rather regulates VSMC phenotypic transition from more differentiated contractile to synthetic by competing with myocardin*-SRF* (serum responce factor) complex for the contractile gene promoters [52]. Thus, *KLF4* is an important player in aortic aneurysm morphogenesis by regulating VSMC phenotypic switching [53]. We suggest that a marked reduction of miR-133a-3p *in vivo* could be associated with upregulated *KLF4* expression in one third of smooth muscle cells in the TAA tissues (Figure 3C). This supposition is supported by previous *in vitro* studies in rodent cell cultures [25,35] showing that VSMC phenotype switch could be regulated by miR-133a-3p via indirect repression of *KLF4*.

This study has some potential limitations: i) In order to thoroughly examine a homogenous etiological category of aneurysms, we have limited our investigation to the sporadic non-syndromic TAA cases. The samples of patients with severe atherosclerosis (calcified or ulcerating plagues), aortitis or phenotypic characteristics of the known genetic syndromes (Marfan, Ehlers Danlos, and other) were excluded, because these features can lead to skewed results. As a consequence of the abovementioned restrictions the total number of samples used in the analysis was limited. ii) Female sample size was small. A larger cohort of female specimens needs to be examined in the future to reliably corroborate sex-specific variances of miRNA signatures in TAA tissues. iii) We profiled miRNA from TAA and non-TAA samples which differed by age. However, the sequencing data was then validated by qRT-PCR performed on larger TAA and non-TAA groups of comparable age. iv) The control group included heart transplant donors, patients who underwent CABG and healthy volunteers. To diminish the impact of such diversity on the outcome of the study, a strict clinical testing was performed on the control group to confirm the normal measurements of ascending aorta.

## 5. Conclusions

Taken together, these observations point to a critical role of aberrant miRNAs expression in promoting TAA via imbalanced repression of *TGF-β* signaling pathway components and following deregulation of *KLF4* transcription axis in vivo. The miRNA-mediated gene expression regulatory networks elucidated herein in clinical samples have paved the ways to further in vitro studies of the miRNAs functions in controlling of VSMC phenotype switch. Moreover, co-expression analysis of selected miRNAs, *KLF4* and VSMC markers inside the cells should be performed in the future to extend our understanding of the miRNA-modulated gene activation shift during TAA development.

## Figures and Tables

**Table 1:** Demographic and clinical characteristics of control and thoracic aortic aneurysm (TAA) patients selected for profiling of microRNA (miRNA) expression.

	Tissue		Plasma		
Variables	non-TAA (*n* = 6)	TAA (*n* = 8)	non-TAA (*n* = 7)	TAA (*n* = 7)	Operated (*n* = 4)
Age, years ± SD	47 ± 5	62 ± 10	54 ± 12	63 ± 11	64 ± 12
Sex, male (%)	4 (67 %)	6 (75 %)	4 (57 %)	5 (71 %)	3 (75 %)
Ascending aortic diameter, mm	36 ± 0.7 *	50 ± 3	35 ± 3	53 ± 5	52 ± 4
Aortic valve stenosis (%)	0 (0 %)	3 (38 %)	1 (14 %)	2 (29 %)	1 (25 %)
Bicuspid aortic valve (%)	0 (0 %)	5 (63 %)	0 (0 %)	4 (57 %)	2 (50 %)
Aortic valve insufficiency (%)	0 (0 %)	5 (63 %)	1 (14 %)	3 (43 %)	1 (25 %)
Hypertension (%)	2 (100 %) *	7 (88 %)	4 (57 %)	6 (86 %)	4 (100 %)
Smokers (%)	2 (100 %) *	1 (13 %)	1 (14 %)	1 (14 %)	0 (0 %)
Diabetes (%)	0 (0 %)	1 (13 %)	0 (0%)	3 (43 %)	1 (25 %)

Notes: * Data is missing from four aorta donors. Operated denotes patient samples collected after aortic surgery.

**Table 2:** A number of miRNAs differentially expressed (fold change > 1.5) in TAA tissue and blood plasma samples compared to non-TAA controls.

Groups	Number of miRNAs	Upregulated	Downregulated
Tissue			
TAA vs. non-TAA	20	15	5
Plasma			
TAA vs. non-TAA	14	3	11
TAA v.s Op	6	4	2
TAA vs. non-TAA + Op	10	2	8

Notes: TAA—Thoracic Ascending Aneurysm; Op—Operated.

**Table 3:** List of differentially expressed miRNAs (selection criteria were fold change > 1.5, *p* value < 0.05 and base mean higher than 10) in TAA aortic tissue samples compared to miRNA expression levels in non-TAA controls.

No.	miRNAs	Fold Change	*p* Value
Upregulated			
1	hsa-miR-10a-3p	2.69	2.05E–06
2	hsa-miR-10a-5p	2.45	8.63E–07
3	hsa-miR-150-5p	2.21	2.05E–05
4	hsa-miR-199b-5p	2.12	1.19E–04
5	hsa-miR-126-5p	1.89	7.95E–04
6	hsa-miR-126-3p	1,88	2.10E–05
7	hsa-miR-139-5p	1.74	7.22E–04
8	hsa-miR-148a-3p	1.71	3.44E–05
9	hsa-miR-10b-5p	1.70	7.78E–04
10	hsa-miR-148a-5p	1.70	0.0112
11	hsa-miR-99a-5p	1.68	1.76E–05
12	hsa-miR-21-5p	1.67	1.10E–03
13	hsa-miR-146a-5p	1.67	0.002
14	hsa-miR-142-3p	1.66	0.020
15	hsa-miR-542-3p	1.64	0.009
Downregulated			
16	hsa-miR-1-3p	−1.59	0.001
17	hsa-miR-133a-3p	−1.64	2.96E–07
18	hsa-miR-1307-3p	−1.68	0.011
19	hsa-miR-9-3p	−1.79	0.021
20	hsa-miR-155-5p	−1.88	7.34E−08

**Table 4:** List of differentially expressed miRNAs (fold change > 1.5, *p* value ≤ 0.05-fold and base mean ≥ 20) in TAA patient blood plasma samples compared to miRNA expression levels in non-TAA controls.

Group	No.	miRNA	Regulation	Fold Change	*p* Value
TAA vs. non-TAA	1	hsa-miR-146b-3p	up	9.11	0.044
	2	hsa-miR-1255b-5p	up	8.87	0.015
	3	hsa-miR-889-3p	up	7.95	0.047
	4	hsa-miR-375-3p	down	–2.38	0.036
	5	hsa–miR-30a-5p	down	–2.54	0.033
	6	hsa-miR-483-3p	down	–2.68	0.015
	7	hsa-miR-23b-3p	down	–2.79	0.017
	8	hsa-miR-140-3p	down	–4.01	0.010
	9	hsa-miR-100-5p	down	–9.17	0.003
	10	hsa-miR-145-5p	down	–17.36	1.44E–04
	11	hsa-miR-143-3p	down	–17.74	3.27E–05
	12	hsa–miR-23b-5p	down	–24.93	0.013
	13	hsa-miR-122-3p	down	–69.32	3.31E–04
	14	hsa-miR-34a-5p	down	–71.95	4.01E–05
TAA vs. Operated	1	hsa-miR-1255b-5p	up	9.7203	0.045
	2	hsa-miR-4732-3p	up	3.9801	0.050
	3	hsa-miR-6803-3p	up	3.4495	0.011
	4	hsa-miR-22-3p	up	2.5198	0.029
	5	hsa-miR-122-3p	down	–18.4085	0.024
	6	hsa-miR-23b-5p	down	–44.7992	0.001
TAA vs. non-TAA & Operated	1	hsa-miR-1255b-5p	up	11.68	0.004
	2	hsa-miR-22-3p	up	1.73	0.034
	3	hsa-miR-375-3p	down	–2.12	0.049
	4	hsa-miR-483-3p	down	–2.29	0.035
	5	hsa-miR-23b-3p	down	–2.36	0.024
	6	hsa-miR-143-3p	down	–3.83	0.012
	7	hsa-miR-145-5p	down	–4.83	0.019
	8	hsa-miR-23b-5p	down	–29.67	0.003
	9	hsa-miR-34a-5p	down	–48.62	6.26E–05
	10	hsa-miR-122-3p	down	–53.67	2.31E–04

## Supplementary Materials

The following are available online at https://www.mdpi.com/2077-0383/8/10/1609/s1. Supplementary File 1. A detailed evaluation of differential miRNA-Seq data of each tissue sample assessed in the present study. Supplementary File 2. A detailed evaluation of differential miRNA-Seq data of each plasma sample assessed in the present study. Supplementary File 3. Dataset for miRNA target analysis. Supplementary Material. Supplementary methods, results, figures and tables.
