# Supplementary material for: Tissue-Specific miRNAs Regulate the Development of Thoracic Aortic Aneurysm: The Emerging Role of KLF4 Network
Source: J Clin Med. 2019 Oct 3;8(10):1609. doi: 10.3390/jcm8101609 (PMC6832203; doi:10.3390/jcm8101609)
Supplement: Supplementary file 1 [file jcm-08-01609-s001.zip › Supplementary_material.pdf]

Tissue-specific miRNAs regulate the development of thoracic aortic aneurysm: the emerging role of KLF4 network

Stasė Gasiulė[1‡] and Vaidotas Stankevičius[1‡], Vaiva Patamsytė[2], Raimundas Ražanskas[1], Giedrius Žukovas[3], Žana Kapustina[4], Diana Žaliaduonyte Pekšienė[5], Rimantas Benetis[2], Vaiva Lesauskaitė[2] and Giedrius Vilkaitis[1]

[1] Institute of Biotechnology, Vilnius University, Vilnius, LT-10257, Lithuania; [2] Institute of Cardiology, Lithuanian University of Health Sciences, Kaunas LT- 50162, Lithuania; [3] Department of Cardiac, Thoracic and Vascular Surgery, Lithuanian University of Health Sciences, Kaunas LT- 50161, Lithuania; [4] Thermo Fisher Scientific Baltics, Vilnius, LT-02241, Lithuania. [5] Department of Cardiology, Lithuanian University of Health Sciences, Kaunas, LT- 50161, Lithuania.

‡ These authors participated equally preparing the manuscript

Correspondence:

Giedrius Vilkaitis, PhD
Institute of Biotechnology
Vilnius University
Sauletekio 7, V305
LT-10257 Vilnius, Lithuania
giedrius.vilkaitis@bti.vu.lt

Vaidotas Stankevicius, PhD
Institute of Biotechnology
Vilnius University
Sauletekio 7, V307
LT-10257 Vilnius, Lithuania
vaidotas.stankevicius@gmc.vu.lt;

SUPPLEMENTARY MATERIAL

SUPLEMENTARY METHODS

Preparation of aortic tissue samples

Tissue samples from ascending aorta aneurysm were obtained during aortic reconstruction surgery (n=17). From each case two samples were taken from the outer curvature of the dilated ascending aorta. The first sample was stored in RNAlater™ solution (Thermo Fisher Scientific) at -80 °C and was used for RNA isolation. The second sample was processed for histology and immunohistochemistry.

Aortic tissue specimens (punch biopsies) from CABG patients (n=35) were taken from ascending aorta at the site of proximal bypass anastomosis. Punches were stored in RNAlater™ solution (Thermo Fisher Scientific) at -80 °C and were used for RNA isolation.

Ascending aorta samples that were left after donor heart transplantation were taken during heart transplantation procedure (n=5). The first sample was stored in RNAlater™ solution (Thermo Fisher Scientific) at -80 °C and was used for RNA isolation. The second sample was processed for histology and immunohistochemistry.

Preparation of blood plasma samples

Blood samples from the patients who underwent aortic reconstructive surgery were taken before operation (n=17) and three months after the surgery (n=4). Blood samples from CABG patients were taken before operation (n=26), while blood samples from healthy volunteers (n=8) and non-operated patients with ascending aortic aneurysm (n=17) were taken after transthoracic echocardiography.

Venosafe™ K2EDTA tubes (Therumo Europe) were used for blood collection and plasma was isolated as described previously (miRNeasy Serum/Plasma Handbook). The level of hemolysis in plasma samples was evaluated measuring optical absorbance at 414 nm (absorbance peak of hemoglobin) using NanoDrop™ 2000 spectrophotometer (ThermoFisher Scientific). In parallel, the expression levels of miR-16 and miR-451a as reliable additional indicators for hemolysis were evaluated in the same samples (described in Quantitative real-time PCR analysis section). Samples were excluded if absorbance A414 values exceeded 0.2 and cycle threshold (Ct) values were below

19 and 22 for miR-451 and miR-16, respectively (**Supplementary table 7**). Plasma samples were stored at –80 °C.

RNA isolation

Total RNA from aortic tissue or blood plasma samples was isolated using mirVana™ Ambion miRNA Isolation Kit (ThermoFisher Scientific) or mirVana™ Paris miRNA Isolation Kit™ (ThermoFisher Scientific), respectively, according to manufacturer's instructions. RNA samples were eluted in pre-heated nuclease-free water, aliquoted and stored at -80°C until further analysis. Total RNA isolated from aortic tissue samples was treated with dsDNase (ThermoFisher Scientific) according to manufacturer's instructions.

cDNA library preparation for Illumina sequencing

cDNA libraries for sequencing were prepared using NEXTflex™ Small RNA-Seq v3 kit (PerkinElmer) according manufacturer's instructions. Briefly, sequent ligation reactions of 3′ and 5′ adapters were performed using 1,6 µg of total RNA or 10,5 µL of each RNA samples isolated from aortic tissues or blood plasma, respectively. The cDNA was amplified 18 or 23 PCR cycles for tissue or plasma cDNA samples, respectively. PCR products were analyzed with Bioanalyzer (Agilent technologies) using DNA high sensitivity chip (**Supplementary Figure 5A**). In order to deplete adapter dimers, plasma cDNA samples were size-selected (150 bp fragment) on a 6 % PAGE and purified according manufacturer's protocol (**Supplementary Figure 5B**). The resulting cDNA libraries were quantified by KAPA™ Library Quantification Kit (Rosche), hybridized to Illumina MiSeq™ flow cell (Illumina) and subsequently sequenced using Illumina MiSeq™ instrument and MiSeq™ Reagent Kit v2 chemistry (Illumina) for 50 sequencing cycles.

miRNA-Seq data assessment

Base calling was performed using CASAVA v1.8 (Illumina) and read sequences were outputted in FASTQ format. Quality control of raw sequences was checked using FastQC v0.11.7. Reads were used when quality retain ≥Q30. Next, raw data of FASTQ files were processed with custom R scripts. Briefly, identical sequences were counted and collapsed, adapter sequences were trimmed and reads containing poly A, T, G or C, shorter than 16 nt and longer than 28 nt were removed. The remaining reads were aligned to human miRNA hairpin sequences from miRBase v22 [1] with Bowtie 1.1.2 [2]. Additional alignment cycles with modified parameters were

performed to include miRNA sequences with 1, 2 or 3 non-template 3'-terminal nucleotides. In addition, any aligned sequence was accepted as mature miRNA if its 5'-terminal mapped to hairpin no further than 1 nt in either direction from the 5' start position of corresponding mature miRNA. Data normalization and differential miRNA expression analyses were performed with R package DeSeq2 [3]. NGS data are available at GEO database (Accession No. GSE122266).

miRNA target analysis

miRNA – mRNA interaction scores were generated by compiling data from eight interaction databases, which use different interaction prediction algorithms. miRNA – mRNA interaction probability values, which were obtained from databases, were converted to percentiles and resulted in values from 0 to 1 (or 0.5 to 1 in case were database contained only set of highly probable interactions). Data without any interaction probability values were assigned equal scores by assessing the ratio of all interactions in corresponding database to the count of interactions in our in-house database. In case of database with experimental data, interactions with strong experimental evidence were given score 3, and interactions with weak experimental evidence were given score 1. Overall, the scores of resulting 11,592,145 interactions were generated by summing values from: RepTar [4], MirTar [5], Miranda [6], PicTar2 [7], MirMap [8], miRTarget3 [9], TargetScan7 [9] and experimental database mirTARbase [10]. Targets exceed score value of 4 were selected for further KEGG pathway enrichment and network analysis, which was performed using Cytoscape v3.6 and ClueGo plugin as described previously [11].

Quantitative real-time PCR analysis

For the miRNA expression analysis, 100 ng or 3 µL of total RNA isolated from aortic tissue or plasma samples were used for the cDNA synthesis as described previously [12]. For mRNA expression analysis, 100 ng of total RNA isolated from aortic tissue samples used for cDNA synthesis with RevertAid™ RT reverse transcriptase (ThermoFisher Scientific) according to manufacturer's instructions. Quantitative real-time PCR analysis was carried out on Rotor-Gene™ 6000 (Corbett Life Science) using SYBR™ Green PCR Master Mix (ThermoFisher Scientific) according to manufacturer's instructions. The miRNA expression levels were normalized to the expression levels of miR-152-3p or mir-185-3p for tissue or plasma samples, respectively. These endogenous miRNAs were selected according to our miRNA-Seq analysis having the lowest

expression difference and deviation between samples. GAPDH was used as endogenous control for the mRNA expression normalization in aortic tissues. The primer sequences used for RT-PCR analysis are shown in **Supplementary Table 8**.

Histology and Immunohistochemistry

Aortic tissue samples were fixed in 10 % neutral buffered formalin solution for 24 hours and underwent a standard paraffin embedding procedure. Three-micron-thick sections were routinely stained with hematoxylin and eosin for histopathological examination of aortic sample.

Immunohistochemical examination was performed on paraffin-embedded donor (N=6), and aneurysm (N=21) aortic tissue sections (3 μm) using EnVision™ FLEX+ visualization system (Agilent, Denmark) according manufacturer's instructions. Additional samples of "punch" biopsies were used to increase control group of KLF4 (N=17) and OPN (N=26) expression analysis. The following primary antibodies were used: KLF4 (ab215036, Abcam; dilution 1:2000), ALK1 (PA5-49803, ThermoFisher Scientific; dilution 1:100), TGFBR1 (PA5-326311, ThermoFisher Scientific; dilution 1:50), Myocardin (SAB4200539, Sigma-Aldrich, USA; dilution 1:100) and osteopontin (DSHB Hybridoma Product MPIIIB10(1); dilution 1:50). Positive control for immunohistochemistry was used according manufacturers' instructions For negative control, primary antibody was omitted from staining procedure and an irrelevant IgG of the same isotype as the primary antibody dilution solution served as negative control: Rabbit IgG Isotype Control (MA5-16384, ThermoFisher Scientific) for KLF4, Rabbit IgG polyclonal Isotype Control (ab37415, Abcam) for ACVRL1, TGFBR1, Myocardin, and Mouse IgG1 Kappa Monoclonal Isotype Control (ab91353, Abcam) for osteopontin (**Supplementary Figure 6).** Stained slides were counterstained with haematoxylin using a Varistain™ Gemini ES automated slide stainer (Thermo, Electron Corporation). Aortic tissue specimens were evaluated using Olympus BX51 Microscope and Image-Pro Plus 7.0 image processing software. KLF4 expression was evaluated by counting KLF4 positive nuclei in five histological fields (0.15 mm^2 each). A semi-quantitative expression of ACVRL1, TGFBR1, and myocardin was carried out by 2 investigators independently assigning grades from 0 (no expression) to 3 (most widespread expression). Final score was calculated as a sum of grades defined by both investigators and ranged from 0 to 6.

Statistical analysis

All statistical analysis was performed using GraphPad v6.0 software. Student's *t* test was used to compare the statistical significance of differences between aneurysmal and non-TAA groups of normally distributed data. Demographic and histological data were assessed with non-parametric Mann-Whitney U test. The differences were assigned as statistically significant when *p* value was lower than 0.05.

SUPPLEMENTARY RESULTS

Overview of miRNA-Seq experimental design

In order to determine miRNAs which expression levels are potentially deregulated in aorta tissue and blood plasma during the formation of TAA, in the present study we evaluated miRNA expression profiles in a learning set of patient tissue and plasma samples (n=32) using Illumina high-throughput miRNA sequencing platform (**Supplementary Figure 7A**). For the tissue analysis, eight TAA samples as well as six aortic non-TAA samples taken from heart transplantation donors (n=4) and aortic biopsies from CABG patients (n=2) were collected (**Table 1**). The four TAA plasma samples were isolated from the same group of patients which were included in the TAA tissue group, thus reducing probable miRNAs variations caused by genetic differences across individual patients. To monitor surgical treatment outcome for changes in miRNAs expression, we also collected and studied plasma samples from the same TAA patients at least 3 months after the surgery. The quality of plasma specimens was evaluated by measuring absorption at 414 nm and RT-qPCR of miR-451a and miR-16-5p (see detailed information in the "Materials and Methods"). A few plasma samples exceeded the permissible hemolysis threshold and were replaced with additional samples (**Supplementary Figure 7A**) to prevent possible contamination of sample with miRNAs from blood cells. Meanwhile, a non-TAA group of seven plasma samples was isolated from independent group of healthy patients.

High-throughput small RNA sequencing of the corresponding samples subsequently generated 80.7 million raw sequencing reads. Following adapter trimming and further filtering steps, approximately 69.1 million of high-quality reads (85.6% of initial raw reads) were retained. The remaining reads ranged from 1.5 to 3.7 million and from 0.99 to 2.5 million per tissue and plasma sample, respectively (**Supplementary Table 9**), and predominantly contained 20-23 nt long sequences which correspond to the size distribution of the mature miRNAs (**Supplementary Figure 7B**). Indeed, around 94% of the filtered reads obtained from aorta tissue sample libraries

were aligned to mature miRNA sequences from miRBase v22 showing a significant library enrichment in small ncRNAs (**Supplementary Figure 7C**). Remarkably, the mapping to miRNA sequences averaged in prevailing 80% of all reads even in challenging plasma sample libraries with very low amounts of RNA. The remaining reads of RNA-Seq datasets from plasma were assigned to the various RNA species including rRNAs, tRNA, lncRNA, other non-coding and coding RNA sequences or human genomic DNA (both averaged in ~7%) indicating a higher diversity of sequence moieties circulating in blood (**Supplementary Figure 7C**; **Supplementary Table 10**). On average 531 and 347 individual human miRNA species were detected in aorta tissue and plasma samples, respectively, demonstrating a higher miRNAs variability and abundance in ascending aorta tissues (**Supplementary Figure 7D**).

SUPPLEMENTARY FIGURES

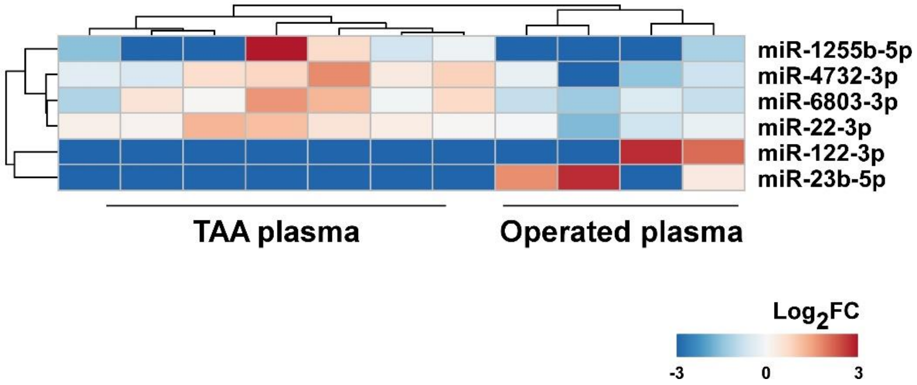

Supplementary Figure 1. Heat map of the expression of six miRNAs, which were significantly deregulated in TAA plasma samples 3 months after aortic surgery compared to miRNA levels in TAA samples before the surgery. Color intensity indicates log-transformed normalized read counts of corresponding miRNA.

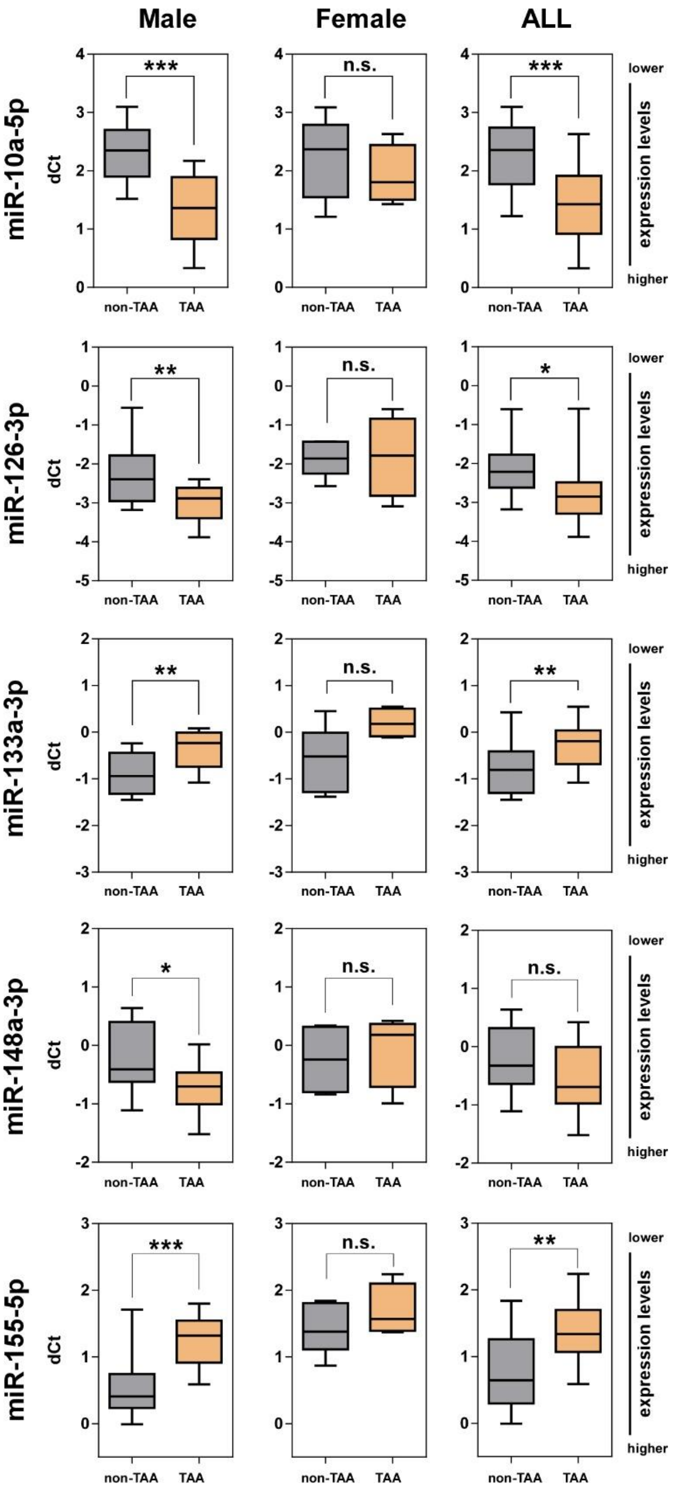

Supplementary Figure 2. Relative expression of miR-10a-5p, miR-126-3p, miR-133a-3p, miR-148a-3p and miR-155-5p in non-TAA and TAA patient tissue samples according to patient's sex. The expression threshold (Ct) values of each miRNA were normalized to miR-152-3p as endogenous control. Lines within boxes indicate normalized cycle threshold (dCt) mean values; whiskers – 5-95 percentile of the relative miRNA expression values. Significance between each group is shown as follows: n.s. – not significant; * $p<0.05$; ** $p<0.01$ and *** $p<0.001$.

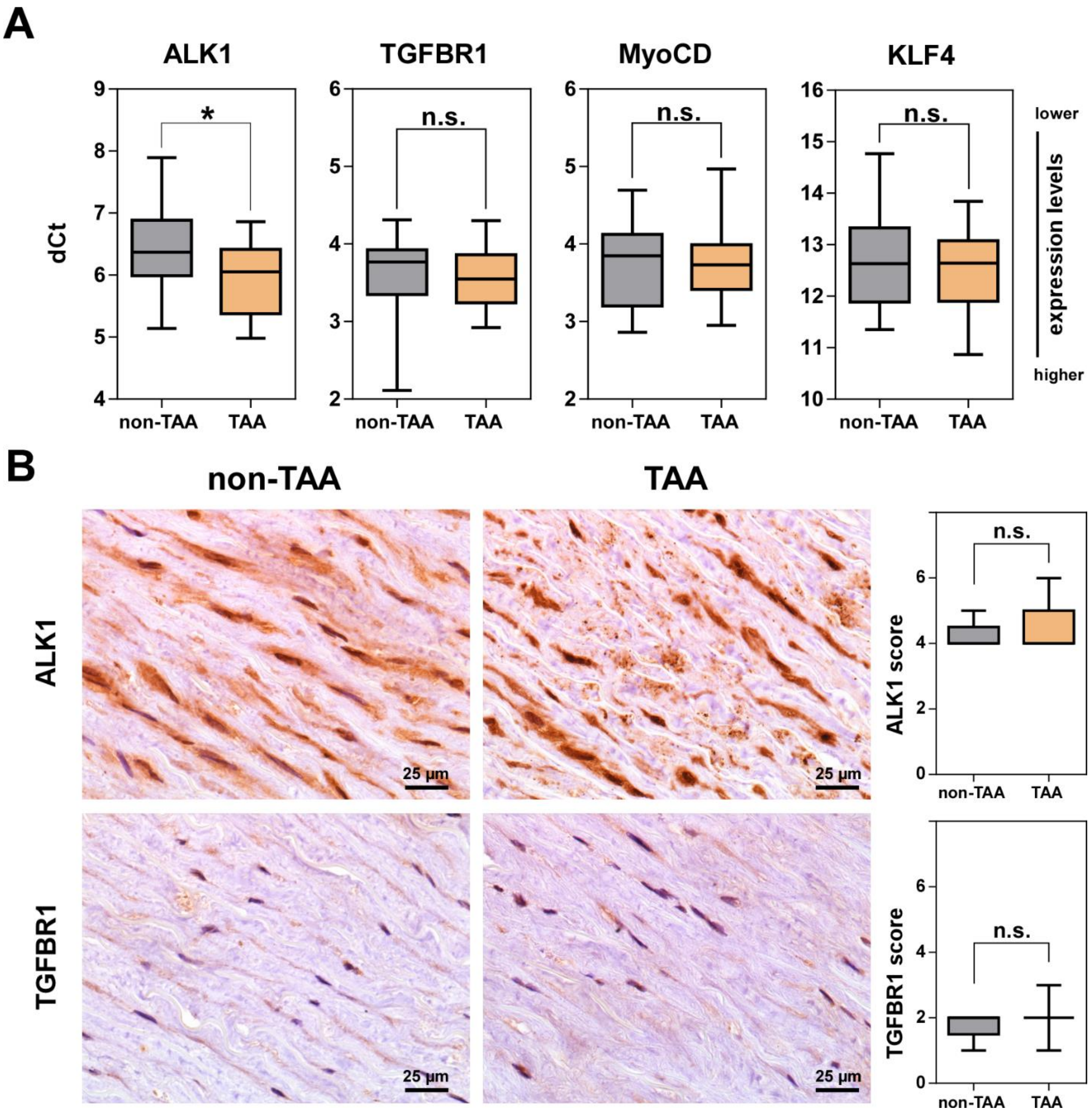

Supplementary Figure 3. A) Relative expression of ALK1, TGFBR1, MyoCD and KLF4 mRNA in non-TAA (n=21) and TAA (n=17) tissue samples. The expression threshold (Ct) values of each gene were normalized to GAPDH expression levels. Lines within boxes indicate normalized cycle threshold (dCt) mean values; whiskers – 5-95 percentile of the relative mRNA expression values. Significance between each group is shown as follows: n.s. – not significant; * p<0.05. **B)** The expression of TGFBR1 and ALK1 was examined by immunohistochemistry (brown) in non-TAA (n=5) and TAA (n=15) tissue samples. The sections were-counterstained-with hematoxylin (blue).

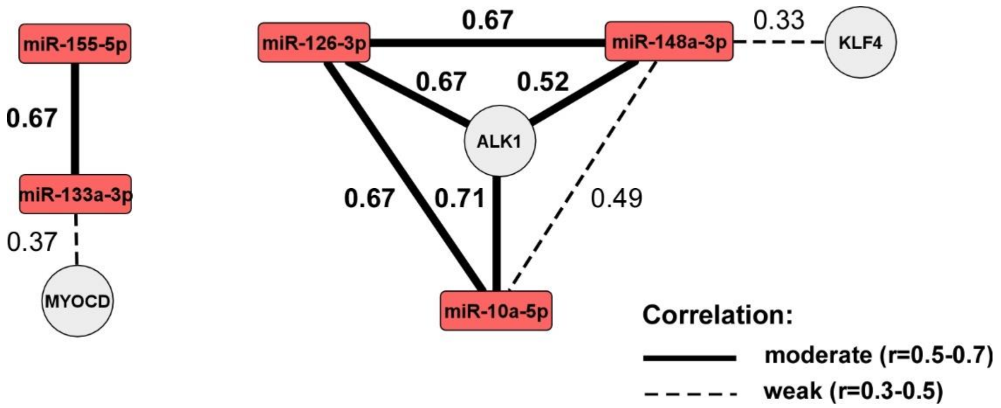

Supplementary Figure 4. Representative co-expression analysis of miRNAs and genes evaluated in non-TAA and TAA tissue samples. Lines represent the strength of the co-expression correlation; numbers – correlation coefficient *r*.

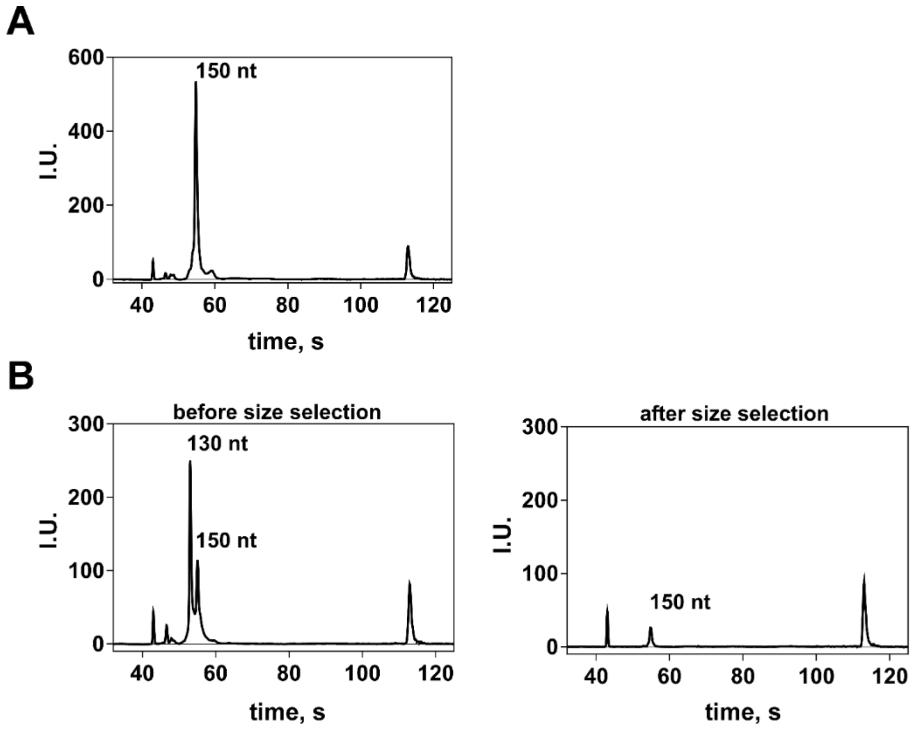

Supplementary Figure 5. Representative images of miRNA-seq libraries prepared from aorta tissue **(A)** and plasma **(B)** samples fractionated using Agilent High Sensitivity DNA Kit. A peak of ~150 nt covers an appropriate miRNA library with adapters. The following PAGE-gel based size selection eliminated ~130 nt peak of the adapter dimers. I.U. – normalized intensity units.

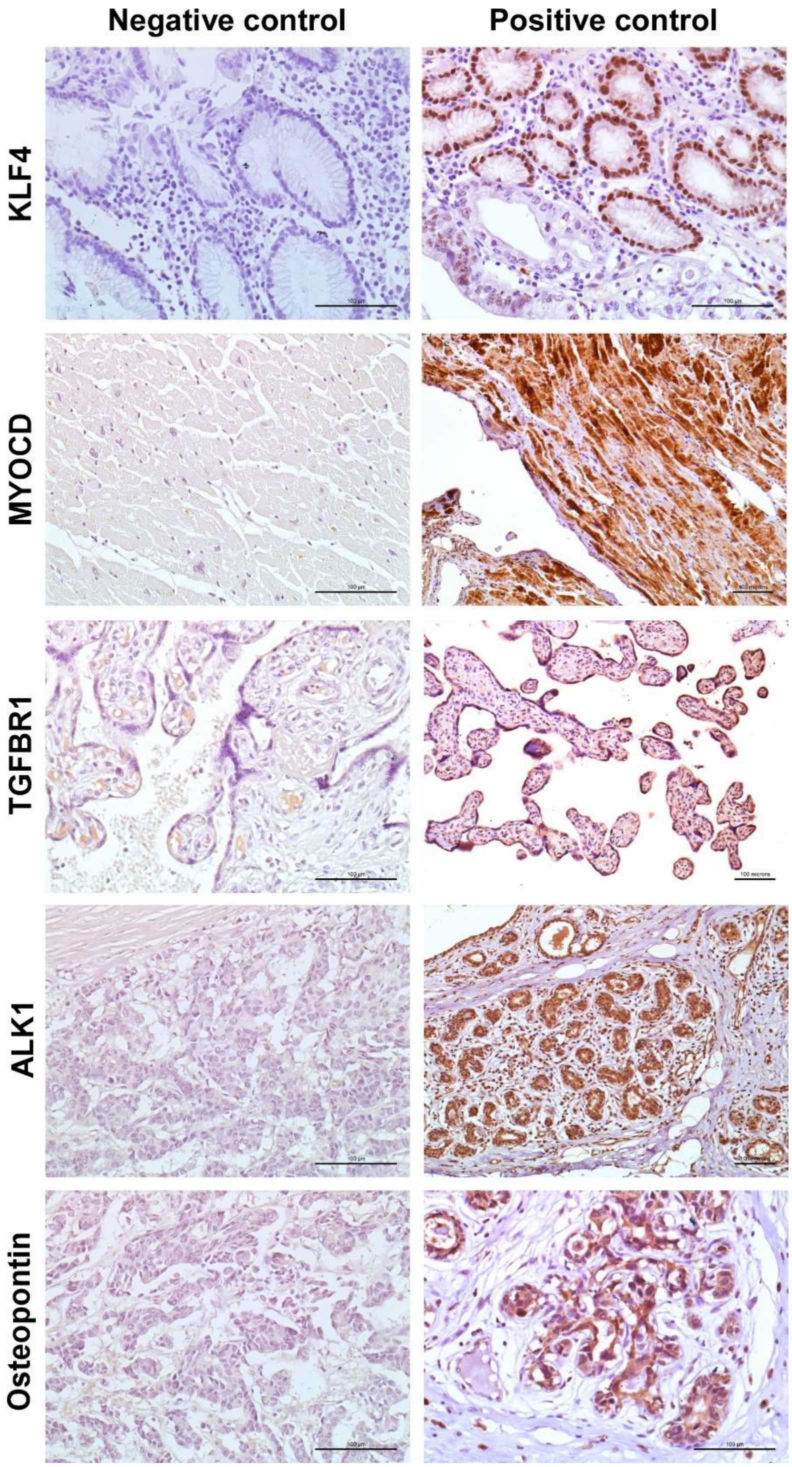

Supplementary Figure 6. Representative negative and positive staining for the expression of KLF4, MYOCD, TGFBR1, ALK1 and osteopontin was examined by immunohistochemistry and visualized with diaminobenzidine (brown). The sections were counterstained with hematoxylin. The expression of each protein was examined in different types of tissue as follows: KLF4 – stomach cancer; MYOCD - myocardium; TGFBR1 - placenta; ALK1 and osteopontin – breast cancer. Bar: 100 µm.

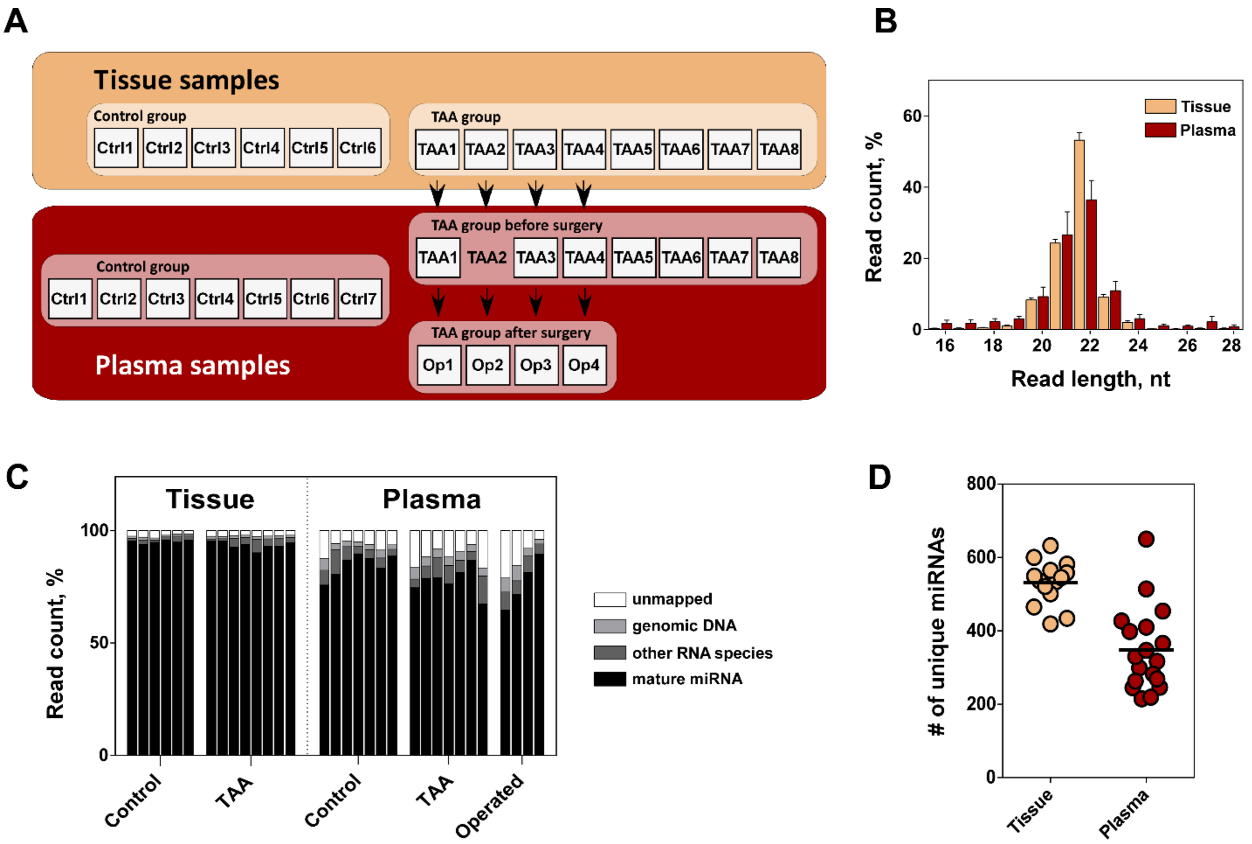

Supplementary Figure 7. Overview of experimental design and miRNA-Seq data analysis checkpoints. **A)** Schematic diagram of miRNA-Seq experiment. Ctrl underlies non-TAA patient samples, TAA – samples collected from patient group diagnosed with thoracic aorta aneurysm before surgery and Op (*operated*) – 3 months after the aortic surgery; **B)** Length distribution of the adapter-trimmed read counts per library within aorta tissue and plasma samples; **C)** A distribution of trimmed 16-28 nt reads by RNA or DNA species within each aorta tissue and plasma sample. Reads, which failed to align to the human genome sequences, are showed as *unmapped*; **D)** Number of unique mature miRNA species identified per aorta tissue and plasma samples.

SUPPLEMENTARY TABLES

Supplementary Table 1. Demographic and clinical characteristics of non-TAA and TAA patients enrolled in this study.

	Tissue			Plasma		
**Variables**	**non-TAA (n=35)**	**TAA (n=17)**	**P value**	**non-TAA (n=34)**	**TAA (n=28)**	**P value**
**Age, years ±SD**	64 ±12	62 ±13	0.610	69 ±12	65 ±12	0.161
**Sex, male (%)**	25 (71 %)	13 (76 %)	0.779	17 (50 %)	21 (75 %)	0.092
**Ascending aortic diameter, mm**	36 ±4*	53 ±6	<0.001	35 ±4	49 ±6	<0.001
**Aortic valve stenosis (%)**	0 (0 %)	5 (29 %)	0.091	4 (12 %)	7 (25 %)	0.379
**Bicuspid aortic valve (%)**	0 (0 %)	11 (65 %)	<0.001	0 (0 %)	11 (39 %)	<0.001
**Aortic valve insufficiency (%)**	10 (29 %)	11 (65 %)	0.042	13 (38 %)	16 (57 %)	0.080
**Hypertension (%)**	30 (100 %)*	14 (82 %)	0.322	29 (85 %)	26 (93 %)	0.460
**Smokers (%)**	6 (20 %)*	3 (18 %)	0.904	4 (11 %)	2 (7 %)	0.857
**Diabetes (%)**	3 (9 %)	2 (12 %)	0.928	3 (9 %)	4 (14 %)	0.718

** Data missing from five donors;*

Supplementary Table 2. miRNA coexpression analysis in aortic non-TAA and TAA tissue samples. Numbers represent correlation coefficient R; colors – positive (red) miRNA expression correlation. ** p<0.01; *** p<0.001.

	**miR-126-3p**	**miR-148a-3p**	**miR-155-5p**	**miR-133a-3p**	**miR-10a-5p**
**miR-126-3p**	x	**0.67*** **	0.11	0.18	**0.67*** **
**miR-148a-3p**		x	-0.17	-0.01	**0.49** **
**miR-155-5p**			x	**0.67*** **	-0.27
**miR-133a-3p**				x	-0.25
**miR-10a-5p**					x

Supplementary Table 3. miRNA coexpression analysis in non-TAA and TAA patient's plasma samples Numbers represent correlation coefficient R; colors – positive (red) or negative (green) miRNA expression correlation. ** p<0.01; ***p<0.001

	**miR-4732-3p**	**miR-483-3p**	**miR-122-3p**	**mir-143-3p**
**miR-4732-3p**	x	-0.22	**-0.33** **	0.05
**miR-483-3p**		x	**0.65*** **	0.24
**miR-122-3p**			x	0.14
**miR-143-3p**				x

Supplementary Table 4. KEGG pathway enrichment analysis of miRNA target genes. The enrichment significance of KEGG categories was calculated by hypergeometric distribution and FDR was evaluated using Bonferroni post-hoc test. KEGG categories were assign as significant when associated with at least 15 genes, FDR<0.05.

KEGG Category	# of genes	FDR
Pathways in cancer	78	8.06E-15
Proteoglycans in cancer	42	1.18E-12
MicroRNAs in cancer	48	4.88E-10
MAPK signaling pathway	46	3.87E-09
PI3K-Akt signaling pathway	50	2.06E-08
AGE-RAGE signaling pathway in diabetic complications	23	1.64E-07
Breast cancer	28	0.000001
Hepatitis B	29	0.000001
Colorectal cancer	20	0.000002
FoxO signaling pathway	25	0.000021
Focal adhesion	30	0.000025
Pancreatic cancer	17	0.000059
Prostate cancer	19	0.000077
Hepatocellular carcinoma	26	0.000109
Bacterial invasion of epithelial cells	16	0.000191
Ras signaling pathway	31	0.000213
Renal cell carcinoma	15	0.000371
TGF-beta signaling pathway	17	0.001202
Fluid shear stress and atherosclerosis	21	0.001202
Signaling pathways regulating pluripotency of stem cells	21	0.001351
Neurotrophin signaling pathway	19	0.001359
Human papillomavirus infection	37	0.001386
Choline metabolism in cancer	17	0.001440
Herpes simplex virus 1 infection	25	0.001466
Endocytosis	30	0.001577
TNF signaling pathway	18	0.001581
Transcriptional misregulation in cancer	25	0.001588
HIF-1 signaling pathway	17	0.002204
Human cytomegalovirus infection	28	0.002352
Small cell lung cancer	16	0.002381
Regulation of actin cytoskeleton	27	0.005855
Measles	19	0.008366
Chagas disease (American trypanosomiasis)	16	0.008675
Rap1 signaling pathway	25	0.008755
Chagas disease (American trypanosomiasis)	15	0.009851
Cellular senescence	21	0.010330
Gastric cancer	20	0.012035
Osteoclast differentiation	18	0.016469
AMPK signaling pathway	17	0.024460
Cell cycle	17	0.024940
Toxoplasmosis	16	0.028260
Apelin signaling pathway	18	0.030748
Toll-like receptor signaling pathway	15	0.033413
Human immunodeficiency virus 1 infection	24	0.033609
Thyroid hormone signaling pathway	16	0.037320
Epstein-Barr virus infection	23	0.040528
mTOR signaling pathway	19	0.043949
Hippo signaling pathway	19	0.000001

Supplementary Table 5. KEGG pathways which were not associated to any other KEGG category.

KEGG Category	# of genes	FDR
MicroRNAs in cancer	48	4.87E-10
Bacterial invasion of epithelial cells	16	0.000109
**TGF-beta signaling pathway**	**17**	**0.000371**
Signaling pathways regulating pluripotency of stem cells	21	0.001202
Endocytosis	30	0.001466
Transcriptional misregulation in cancer	25	0.001581
Small cell lung cancer	16	0.002352
Cell cycle	17	0.024460
Apelin signaling pathway	18	0.028260
Hippo signaling pathway	19	0.043949

Supplementary Table 6. Coexpression analysis of genes and miRNAs in aortic tissue. Numbers represent correlation coefficient R; colors – positive (red) miRNA expression correlation. * p<0.05 ** p<0.01; ***p<0.001

	KLF4	MYOCD	TGFR1	ALK1
**miR-126-3p**	0.27	-0.04	-0.14	**0.67*** **
**miR-148a-3p**	**0.33***	0.21	0.03	**0.52****
**miR-155-5p**	-0.22	0.21	-0.08	-0.28
**miR-133a-3p**	-0.22	**0.37 ***	0.09	0.05
**miR-10a-5p**	0.20	0.03	0.03	**0.71*** **

Supplementary Table 7. Absorbance levels at 414 nm and Ct values for miR-16-5p and miR-451a from plasma samples which were used in miRNA-Seq analysis.

Samples	Absorbance, 414 nm	Ct, miR-16-5p	Ct, miR-451a
Positive control	0.36	21.13	17.03
**Non-TAA 1**	0.11	23.21	21.35
**Non-TAA 2**	0.12	23.93	24.39
**Non-TAA 3**	0.10	22.97	20.94
**Non-TAA 4**	0.13	24.00	23.31
**Non-TAA 5**	0.17	24.88	23.24
**Non-TAA 6**	0.20	25.43	24.96
**Non-TAA 7**	0.20	24.94	23.04
**TAA 1**	0.13	22.93	19.40
**TAA 3**	0.20	23.40	21.77
**TAA 4**	0.20	23.02	20.88
**TAA 5**	0.16	22.03	19.21
**TAA 6**	0.16	22.57	20.76
**TAA 7**	0.14	23.67	19.59
**TAA 8**	0.13	23.85	19.62
**Operated 1**	0.13	22.88	20.15
**Operated 2**	0.17	22.34	22.36
**Operated 3**	0.15	22.73	19.97
**Operated 4**	0.18	24.03	20.83
Negative control	0.18	24.28	19.92

Operated denotes to samples collected 3 months after aortic reconstructive surgery

Supplementary Table 8. List of primer sequences used for RT-qPCR analysis.

**miRNA RT primers**	
**miR-185-5p**	5‘-GTCGTATCCAGTGCAGGGTCCGAGGTATTCGCACTGGATACGACTCAGGAAC
**miR-122-3p**	5‘-GTCGTATCCAGTGCAGGGTCCGAGGTATTCGCACTGGATACGACATTTAGTG
**miR-483-3p**	5‘-GTCGTATCCAGTGCAGGGTCCGAGGTATTCGCACTGGATACGACAAGACGGG
**miR-4732-3p**	5‘-GTCGTATCCAGTGCAGGGTCCGAGGTATTCGCACTGGATACGACCAGAACAG
**miR-152-3p**	5‘-GTCGTATCCAGTGCAGGGTCCGAGGTATTCGCACTGGATACGACCCAAGTTC
**miR-126-3p**	5‘-GTCGTATCCAGTGCAGGGTCCGAGGTATTCGCACTGGATACGACCGCATT
**mir-451a**	5‘-GTCGTATCCAGTGCAGGGTCCGAGGTATTCGCACTGGATACGACAACTCA
**miR-133a-3p**	5’-GTCGTATCCAGTGCAGGGTCCGAGGTATTCGCACTGGATACGACCAGCTGGT
**miR-10a-5p**	5'- GCTTGTCGGTTAAACACTGTCACAAATTCG
**miR-155-5p**	5‘-CCAGAAACCGATCAGAGTGTACCCCTATCA
**miR-148a-3p**	5‘-CCGTTCACGATCCAAAGACACAAAGTTCT
**miR-143-3p**	5‘-TCGTCGAGATAAGCTGTGTGTGAGCTACA
**mir-16-5p**	5‘-CCTTTGAGGTTGGTACTACGGCGCCAATA
**miRNA RT-qPCR primers**	
**mir-185-5p-FW**	5‘-GCGTGGAGAGAAAGGCAGT
**mir-483-3p FW**	5‘-GCGTCACTCCTCTCCTCC
**mir-4732-3p FW**	5‘-GGCCCTGACCTGTCCT
**mir-152-3p FW**	5‘-CGCGTCAGTGCATGACAGA
**mir-126-3p FW**	5‘-CGCGTCGTACCGTGAGTAAT
**mir-122-3p- FW**	5‘-CCGCGAACGCCATTATCACA
**mir-133a-3p FW**	5‘-GCGTTTGGTCCCCTTCAAC
**mir-10a-5p FW**	5'- AGGCTTGTCGGTTAAACACTGT
**mir-10a-5p REV**	5'- CCGGTACTACCCTGTAGATCCG
**mir-143-3p FW**	5‘-GCTCGTCGAGATAAGCTGTGTG
**mir-143-3p REV**	5‘-GTTCGCTGAGATGAAGCACTG
**mir-155-5p FW**	5‘-TTCCAGAAACCGATCAGAGTGT
**mir-155-5p REV**	5‘-CGCCATGTTTAATGCTAATCGTGA
**mir-16-5p FW**	5‘-ACCTTTGAGGTTACTACGG
**mir-16-5p REV**	5‘-GTGCAGTAGCAGCACGTAAAT
**mir-451a FW**	5‘-CGGCGAAACCGTTACCATTAC
**Universal REV**	5‘-GTGCAGGGTCCGAGGT
**mRNA RT-qPCR primers**	
**GAPDH FW**	5‘-GGAGCGAGATCCCTCCAAAAT
**GAPDH REV**	5‘-GGCTGTTGTCATACTTCTCATGG
**KLF4 FW**	5‘-CAGCTTCACCTATCCGATCCG
**KLF4 REV**	5‘-GACTCCCTGCCATAGAGGAGG
**MYOCD FW**	5‘-ACGGATGCTTTTGCCTTTGAA
**MYOCD REV**	5‘-AACCTGTCGAAGGGGTATCTG
**ALK1 FW**	5‘-CTCACAGGGCAGCGATTACC
**ALK1REV**	5‘-CCAGATGTCAGTCCACTTGTAGG
**TGFR1 FW**	5‘-CACAGAGTGGGAACAAAAAGGT
**TGFR1 REV**	5‘-CCAATGGAACATCGTCGAGCA

Supplementary Table 9. Summarized miRNA-Seq data statistics for each library.

Sample	Raw reads	Trimmed reads	Reads mapped to pri-miRNA	miRNA read count, %	Number of unique miRNAs
**Tissue samples**					
**Non-TAA 1**	3,346,505	3,137,759	2,997,508	95.53	534
**Non-TAA 2**	3,235,644	2,949,897	2,772,745	93.99	536
**Non-TAA 3**	1,905,149	1,674,889	1,588,383	94.84	501
**Non-TAA 4**	3,327,187	2,828,316	2,712,639	95.92	565
**Non-TAA 5**	3,605,727	3,075,178	2,918,211	94.90	582
**Non-TAA 6**	4,146,013	3,788,589	3,630,021	95.81	600
**TAA 1**	3,193,154	2,686,332	2,561,472	95.35	419
**TAA 2**	2,935,573	2,564,224	2,447,226	95.44	465
**TAA 3**	1,889,708	1,544,150	1,432,645	92.79	434
**TAA 4**	3,085,454	2,434,653	2,287,036	93.95	632
**TAA 5**	1,881,213	1,588,459	1,432,674	90.19	558
**TAA 6**	1,929,822	1,676,213	1,562,601	93.22	549
**TAA 7**	2,089,178	1,813,848	1,690,383	93.19	521
**TAA 8**	2,568,962	2,324,013	2,201,407	94.72	545
**Plasma samples**					
**Non-TAA 1**	2,043,485	1,620,343	1,228,453	75.81	317
**Non-TAA 2**	2,952,040	2,400,962	1,934,959	80.59	427
**Non-TAA 3**	3,037,317	2,568,221	2,233,656	86.97	454
**Non-TAA 4**	2,669,924	2,355,714	2,113,387	89.71	514
**Non-TAA 5**	2,660,301	2,466,404	2,161,804	87.65	299
**Non-TAA 6**	2,570,339	2,276,003	1,897,345	83.36	245
**Non-TAA 7**	2,030,078	1,797,423	1,594,747	88.72	215
**TAA 1**	2,406,097	1,943,187	1,451,897	74.72	366
**TAA 3**	2,399,452	2,153,562	1,697,756	78.83	410
**TAA 4**	1,315,159	1,107,591	875,088	79.01	398
**TAA 5**	1,259,406	995,371	759,846	76.35	246
**TAA 6**	2,144,859	1,909,938	1,555,412	81.44	263
**TAA 7**	2,219,607	1,958,665	1,700,072	86.80	219
**TAA 8**	2,733,052	2,414,650	1,627,917	67.42	282
**Operated 1**	2,940,926	1,733,265	1,120,308	64.64	330
**Operated 2**	2,292,600	1,874,941	1,344,119	71.69	269
**Operated 3**	1,330,345	1,103,461	898,694	81.44	347
**Operated 4**	2,561,525	2,302,498	2,062,228	89.56	650

Operated denotes to samples collected 3 months after aortic reconstructive surgery.

Supplementary Table 10. Summarized miRNA-Seq data alignment statistics for each library.

	Mapped to, %											
	miRNAs	lncRNAs	piRNAs	tRNAs	rRNAs	snoRNAs	snRNAs	Other ncRNAs	Coding RNAs	Human genome DNA	Virus DNA	Unmapped
**Tissue samples**												
**Non-TAA 1**	95,53	0,28	0,15	0,06	0,36	0,01	0,00	0,03	0,09	0,91	0,09	2,49
**Non-TAA 2**	93,99	0,37	0,25	0,10	0,79	0,01	0,01	0,04	0,14	1,24	0,10	2,96
**Non-TAA 3**	94,84	0,27	0,21	0,07	0,29	0,02	0,00	0,03	0,12	0,76	0,13	3,26
**Non-TAA 4**	95,91	0,29	0,45	0,08	0,29	0,02	0,00	0,03	0,10	0,69	0,11	2,03
**Non-TAA 5**	94,90	0,55	0,38	0,13	1,32	0,02	0,02	0,06	0,15	0,82	0,12	1,53
**Non-TAA 6**	95,81	0,40	0,13	0,09	0,95	0,01	0,01	0,04	0,15	0,85	0,09	1,47
**TAA 1**	95,35	0,30	0,10	0,05	0,36	0,02	0,00	0,03	0,09	1,07	0,08	2,55
**TAA 2**	95,44	0,25	0,16	0,05	0,40	0,01	0,00	0,03	0,10	0,85	0,09	2,62
**TAA 3**	92,78	0,74	1,00	0,30	1,44	0,03	0,01	0,06	0,26	0,93	0,12	2,33
**TAA 4**	93,94	0,73	0,34	0,15	1,39	0,03	0,01	0,08	0,18	1,00	0,12	2,03
**TAA 5**	90,19	0,72	2,97	0,26	1,67	0,05	0,02	0,06	0,20	1,14	0,13	2,59
**TAA 6**	93,22	0,73	0,17	0,14	1,76	0,03	0,01	0,08	0,21	1,11	0,14	2,39
**TAA 7**	93,19	0,73	0,44	0,14	1,87	0,02	0,02	0,06	0,21	0,98	0,12	2,22
**TAA 8**	94,72	0,52	0,15	0,11	1,26	0,02	0,01	0,05	0,16	0,87	0,10	2,03
**Plasma samples**												
**Non-TAA 1**	75,81	2,40	0,23	0,18	2,97	0,01	0,03	0,49	0,27	4,50	0,77	12,34
**Non-TAA 2**	80,59	6,87	0,57	0,18	2,06	0,01	0,06	0,86	0,23	2,34	0,48	5,75
**Non-TAA 3**	86,97	4,56	0,32	0,12	0,66	0,01	0,04	0,42	0,11	1,83	0,26	4,70
**Non-TAA 4**	89,71	1,27	0,22	0,15	1,11	0,01	0,06	0,24	0,16	1,76	0,31	5,00
**Non-TAA 5**	87,65	1,54	0,26	0,16	1,33	0,01	0,06	0,31	0,15	1,92	0,36	6,25
**Non-TAA 6**	83,36	1,23	0,34	0,28	2,07	0,02	0,03	0,22	0,28	2,87	0,65	8,64
**Non-TAA 7**	88,72	1,00	0,26	0,13	1,06	0,01	0,07	0,27	0,20	1,79	0,46	6,03
**TAA 1**	74,72	0,88	0,11	0,06	2,20	0,01	0,06	0,16	0,23	4,32	0,85	16,40
**TAA 3**	78,83	2,07	0,25	0,26	2,12	0,00	0,01	0,48	0,25	3,56	0,50	11,67
**TAA 4**	79,01	4,37	0,42	0,26	3,21	0,01	0,04	0,49	0,24	3,29	0,59	8,07
**TAA 5**	76,34	2,88	0,25	0,15	4,27	0,02	0,03	0,28	0,27	3,30	0,71	11,50
**TAA 6**	81,44	1,65	0,64	0,43	1,93	0,02	0,05	0,32	0,34	3,43	0,59	9,16
**TAA 7**	86,80	1,42	0,21	0,15	1,42	0,00	0,06	0,29	0,27	2,56	0,52	6,30
**TAA 8**	67,42	5,73	0,49	0,26	4,89	0,00	0,07	0,77	0,21	3,08	0,53	16,54
**Operated 1**	64,64	2,76	0,25	0,12	3,97	0,00	0,09	0,49	0,31	5,59	0,91	20,87
**Operated 2**	71,69	1,39	0,23	0,22	3,57	0,00	0,06	0,18	0,38	5,77	0,89	15,62
**Operated 3**	81,44	2,62	0,23	0,27	2,78	0,01	0,07	1,17	0,25	3,11	0,48	7,57
**Operated 4**	89,56	2,08	0,25	0,20	1,31	0,01	0,04	0,42	0,15	1,74	0,41	3,83

References

1. Kozomara, A. and S. Griffiths-Jones, *miRBase: annotating high confidence microRNAs using deep sequencing data.* Nucleic Acids Res, 2014. **42**(Database issue): p. D68-73.
2. Langmead, B., et al., *Ultrafast and memory-efficient alignment of short DNA sequences to the human genome.* Genome Biol, 2009. **10**(3): p. R25.
3. Love, M.I., W. Huber, and S. Anders, *Moderated estimation of fold change and dispersion for RNA-seq data with DESeq2.* Genome Biol, 2014. **15**(12): p. 550.
4. Elefant, N., et al., *RepTar: a database of predicted cellular targets of host and viral miRNAs.* Nucleic Acids Res, 2011. **39**(Database issue): p. D188-94.
5. Hsu, J.B., et al., *miRTar: an integrated system for identifying miRNA-target interactions in human.* BMC Bioinformatics, 2011. **12**: p. 300.
6. Enright, A.J., et al., *MicroRNA targets in Drosophila.* Genome Biol, 2003. **5**(1): p. R1.
7. Krek, A., et al., *Combinatorial microRNA target predictions.* Nat Genet, 2005. **37**(5): p. 495-500.
8. Vejnar, C.E. and E.M. Zdobnov, *MiRmap: comprehensive prediction of microRNA target repression strength.* Nucleic Acids Res, 2012. **40**(22): p. 11673-83.
9. Wang, X., *Improving microRNA target prediction by modeling with unambiguously identified microRNA-target pairs from CLIP-ligation studies.* Bioinformatics, 2016. **32**(9): p. 1316-22.
10. Chou, C.H., et al., *miRTarBase update 2018: a resource for experimentally validated microRNA-target interactions.* Nucleic Acids Res, 2018. **46**(D1): p. D296-D302.
11. Bindea, G., et al., *ClueGO: a Cytoscape plug-in to decipher functionally grouped gene ontology and pathway annotation networks.* Bioinformatics (Oxford, England), 2009. **25**(8): p. 1091-1093.
12. Butkytė, S., et al., *Splicing-dependent expression of microRNAs of mirtron origin in human digestive and excretory system cancer cells.* Clinical Epigenetics, 2016. **8**(1): p. 33.
